# Spray-dried pH-sensitive chitosan microparticles loaded with *Mycobacterium bovis* BCG intended for supporting treatment of *Helicobacter pylori* infection

**DOI:** 10.1038/s41598-024-55353-6

**Published:** 2024-02-27

**Authors:** Weronika Gonciarz, Marek Brzeziński, Weronika Orłowska, Paweł Wawrzyniak, Artur Lewandowski, Vedha Hari B. Narayanan, Magdalena Chmiela

**Affiliations:** 1 Department of Immunology and Infectious Biology, Institute of Microbiology, Biotechnology and Immunology, Faculty of Biology and Environmental Protection, University of Lodz, Banacha 12/16, 90-237 Lodz, Poland; 2 Centre of Molecular and Macromolecular Studies, Polish Academy of Sciences, Sienkiewicza 112, 90-636 Lodz, Poland; 3 Department of Environmental Engineering, Faculty of Process and Environmental Engineering, Lodz University of Technology, Stefana Zeromskiego 116, 90-924 Lodz, Poland; 4 Pharmaceutical Technology Laboratory, #214, ASK-II, School of Chemical and Biotechnology, SASTRA Deemed University, Thanjavur, Tamil Nadu 613401 India

**Keywords:** Spray-dried chitosan, Microparticles, BCG, Encapsulation, Biodistribution, *H. pylori*, Biomaterials - vaccines, Drug delivery, Drug discovery, Antimicrobials

## Abstract

Gram-negative spiral-shaped *Helicobacter pylori* (*Hp*) bacteria induce the development of different gastric disorders. The growing resistance of *Hp* to antibiotics prompts to search for new therapeutic formulations. A promising candidate is *Mycobacterium bovis* BCG (BCG) with immunomodulatory properties. Biodegradable mucoadhesive chitosan is a good carrier for delivering BCG mycobacteria to the gastric mucosal environment. This study aimed to show whether BCG bacilli are able to increase the phagocytic activity of *Cavia porcellus*—guinea pig macrophages derived from the bone marrow towards fluorescently labeled *Escherichia coli*. Furthermore, to encapsulate live BCG bacilli, in spray-dried chitosan microparticles (CHI-MPs), and assess the pH-dependent release of mycobacteria in pH conditions mimicking gastric (acidic) or gut (alkaline) milieu. Microparticles (MPs) were made of chitosan and coated with Pluronic F-127-(Plur) or *N*-Acetyl-d-Glucosamine-(GlcNAc) to increase the MPs resistance to low pH or to increase anti-*Hp* effect, respectively. Spray-drying method was used for microencapsulation of live BCG. The biosafety of tested CHI-MPs has been confirmed using cell models in vitro and the model of guinea pig in vivo. The CHI-MPs loaded with BCG released live mycobacteria at pH 3.0 (CHI-GlcNAc-MPs) or pH 8.0. (CHI-Plur-MPs). The CHI-MPs loaded with live BCG can be used for *per os* inoculation of *Cavia porcellus* to check the effectiveness of delivered mycobacteria in increasing anti-*H. pylori* host response.

## Introduction

The challenge in pharmaceutics is to encapsulate bioactive substances in an appropriate carrier and their release in the target compartment that ensures a therapeutic activity. For this purpose, different drug delivery systems (DDS) with various modifications have been established^1^. For instance, such DDS can be composed of biosafe chitosan, which is a cationic polysaccharide polymer obtained from chitin by alkaline deacetylation. It contains the *N*-acetyl-d-glucosamine (GlcNAc) and d-glucosamine (Glc) connected by (1,4)-β-glycosidic bound and –OH and –NH_2_ groups, which determine its mucoadhesive properties and its solubility in acidic pH^2–4^. Mucin glycoproteins contain components (proline, threonine, and serine), interacting with Glc and GlcNAc fucose or sialic acid^5^. Moreover, chitosan due to its chemical properties has been considered for usage in industry, medicine, and pharmacy^6–8^. Various studies were devoted to obtaining different materials containing chitosan, including microspheres, nanoparticles, gels, films, or fibers^8–10^. In addition, it can be also used for the development of carriers delivering drugs or bioactive substances to the gastric mucosa for the treatment of *H. pylori* infection. Gastric colonization with *H. pylori* provokes chronic inflammation, which may result in ulceration of the stomach or duodenum, or cancer development^11,12^. These bacteria colonize the human stomach or duodenum in the range of 50–90%. It has been shown that *H. pylori* increase an oxidative stress in gastric epithelial cells, which results in upregulation of apoptosis and gastric barrier dysfunction^13–16^. Furthermore, the activity of different immune cells: phagocytes^17–19^, natural killer (NK) cells^20^, or T lymphocytes^21^ is downregulated in response to *H. pylori* roods or components of these bacteria. In recent years the resistance of *H. pylori* to antibiotics used for the treatment of infected patients (clarithromycin, amoxicillin, levofloxacin or metronidazole) has grown^22–24^. This situation prompts the development of novel therapeutic formulations. Therefore, we hypothesized that *Mycobacterium bovis* (*M. bovis*) can be an active ingredient of encapsulate in the chitosan-based DDS against *H. pylori*. *M. bovis* bacilli are included in the anti-tuberculous Bacillus Calmette-Guerin (BCG) vaccine. It has been shown that BCG mycobacteria may also induce cross-reactive immune response against other bacterial or fungal pathogens such as *Staphylococcus aureus* and *Candida albicans.* Moreover, it may also influence the course of infections caused by respiratory syncytial virus and Sars-Cov-2^25–27^. Vaccine mycobacteria have been used due to their immunomodulatory activity for the treatment of bladder cancer improving the anti-tumor immune response of the host^28^. It has been suggested that mycobacteria, which are internalized by tumor cells due to binding with fibronectin induce cell apoptosis^28^. It was shown that priming and/or restimulation of human THP-1 monocytes or THP-1 derived macrophages with onco-BCG formulation resulted in increased phagocytic activity of these cells towards fluorescently labelled *Escherichia coli* (*E. coli*). It was accompanied by elevated deposition of cell surface receptors (CD11b, CD11d, CD18, CD14, sCD14) involved in phagocytosis, and with the production of macrophage chemotactic protein-1 (MCP-1)^29^.

In the present study, we aim to use macrophages obtained from the bone marrow of *Cavia porcellus*, as primary cells, to evaluate their phagocytic activity after priming and/or restimulation with *M. bovis* BCG. For the first time, spray-drying mucoadhesive chitosan microparticles (CHI-MPs) sensitive to acidic/alkaline pH were used for microencapsulation of live *M. bovis* BCG bacilli. The obtained CHI-BCG-MPs are dedicated to enhancing the immune response towards *H. pylori* or potentially revealing direct anti-*H. pylori* activity. The MPs modifications with Pluronic F-127 or GlcNAc have been applied to increase acidic pH stability and mucoadhesive properties. The *M. bovis* survival and release from MPs have been evaluated.

## Materials and methods

### Reagents

Chitosan (medium molecular weight, 75–85% deacetylated), *N*-Acetyl-d-Glucosamine (GlcNAc), Pluronic F-127 (Plur), and acetic acid tablets were purchased from Sigma Aldrich (Sigma Aldrich, Darmstadt, Germany).

### Equipment

The structural investigations of CHI/GlcNAc/Plur were made by Solid-state Cross-Polarization Magic Angle Spinning Carbon-13 Nuclear Magnetic Resonance (CP/MAS-NMR) using Bruker-Avance AV 400 spectrometer (Bruker, Zurich, Switzerland) equipped with a MAS probe of 4 mm dual-resonance broad-band. To perform analysis, approximately 100 mg desired substance was positioned into the spinner and introduced into the center of the magic field. The NMR spectra were documented at the frequency of 8 kHz at 295 K (accompanied by CP and MAS). A whole of over 1000 scans was chosen for a single spectrum at a recycle interval of 2 s.

Fourier-transform infrared spectroscopy (FTIR) spectra were coupled with Attenuated Total Reflectance (ATR). To perform IR measurements, a Nicolet 6700 spectrometer equipped with a deuterated triglycine sulphate (DGTS) detector was employed. The spectra were obtained by adding 64 scans at a 2 cm^−1^ resolution. Both blank and *M. bovis* BCG-loaded microparticles were analyzed.

Differential scanning calorimetry (DSC) was made on a DSC 2920 modulated TA Instruments under nitrogen at a heating and cooling rate of 10 °C/min. To perform analysis, the temperature and heat flow must be calibrated with indium. The DSC was performed for blank particles. Thermogravimetric analysis (TGA) is a method in which a nitrogen atmosphere is required, and the samples are heated from 25 to 600 °C at a heating rate of 20 °C min^−1^ using a Hi-Res TGA 2950 thermogravimetric analyzer (TA Instruments).

The morphology of spray-dried MPs was analyzed by Scanning Electron Microscopy (SEM). The JEOL JSH 5500 LV microscope operating in high-vacuum mode at the accelerated voltage of 10 kV was used to acquire images. The samples need to be fixed onto carbon adhesive tape covered with a conductive gold layer and studied in a low vacuum.

### Preparation of chitosan microparticles by spray drying technique

The chitosan microparticles—CHI MPs were developed by spray drying technique using the Buchi mini spray dryer (B-290, Buchi mini spray dryer, Buchi, Switzerland) with the spray-drying parameters similar to the conditions described for chitosan particles, as shown by Vedha Hari et al.^30^. Polymers and sugar were dissolved in 1% (v/v) acetic acid to prepare a homogenous solution, whereas, for *M. bovis* BCG-loaded microparticles, the ingredients were mixed until the appropriate suspension was achieved. The number of mycobacteria for each sample was equal to 1 mL  *M*. *bovis* BCG (SYNTHAVERSE S.A. Lublin, Poland) 3 × 10^8^ colony forming units—CFU/mL and the bacteria were fluorescently labelled with BacLight (ThermoFisher, Waltham, USA). Subsequently, spray-dried microparticles were collected via cyclone and stored in a desiccator.

The different microparticles are composed of solely (1) chitosan, (2) chitosan and GlcNAc, (3) chitosan and Plur. Subsequently, these three formulations were used to encapsulate *M. bovis* BCG during the spray-drying process.

### Evaluation of the viability of *M. bovis* BCG encapsulated in chitosan microparticles

CHI, CHI-Plur and CHI-GlcNAc solutions were prepared as described above. Then, 1 mL of *M. bovis* onco-BCG 3 × 10^8^ CFU/mL was added to each type of solution, previously labelled with BacLight fluorescent dye tracer for 30 min at room temperature, which penetrates only into living cells, as previously described^29^. The labelled bacteria were then added to the prepared CHI solutions, and spray-dried as described above. Then, the efficiency of the spray drying process was calculated and the percentage of *M. bovis* BCG bacilli, which survived in 1 mg of individual CHI MPs, was calculated based on the intensity of green fluorescence of labelled mycobacteria, based on using the previously prepared standard curve of labelled bacilli in the range of 1 × 10^8^^−1^ CFU/mL (for each pH separately). Fluorescence intensity was measured using a multimode microplate reader SpectraMaxi3 (Molecular Devices, San Jose, CA, USA), at wavelength: excitation 495 nm, emission 519 nm. In addition, CHI-MPs loaded with mycobacteria were visualized under a fluorescence microscope (Zeiss, Axio Scope.A1, Zeiss, Jena, Germany) at wavelength: excitation 495 nm, emission 519 nm, at 20×, 100× magnification (with immersion). Four independent experiments were carried out in triplicate for each experimental variant. The viability of mycobacteria encapsulated in CHI-MPs was also assessed by plating on Lowenstein–Jensen agar according to the routine procedure. The growth of bacteria at 37 °C in aerobic conditions was controlled for six weeks.

### The assessment of *M. bovis* BCG release from chitosan microparticles in vitro

To determine the release of live *M. bovis* onco-BCG from MPs in alkaline pH = 8.0, acidic pH = 3.0 or neutral pH = 7.2, 100 µL of CHI-MPs (10 mg/mL) loaded with live *M. bovis* BCG previously labelled with BacLight were transferred into the wells of the 96-well plate. The absorbance was measured every 10 min for each pH variant in the wavelength: excitation 495 nm, emission 519 nm, at a time appropriate for the pH of the solution (absorbance was determined based on the stability of the dye in different pH ranges). Free *M. bovis* BCG suspended in PBS pH = 7.2, PBS pH = 3.0 or PBS pH = 8.0 were used as a control. The number of mycobacteria released from CHI MPs (in 1 mg) at the tested pH was calculated based on the previously prepared standard curve of labelled bacilli in the range of 1 × 10^8^^–1^ CFU/mL. The PBS was adjusted to the desired pH using 0.1% HCl or 0.1% NaOH. Four independent experiments were carried out in triplicate for each experimental variant.

### Cell cultures and exposure conditions

The L929 mouse fibroblasts (purchased in LGC Standards, Middlesex, UK), *Cavia porcellus* (guinea pig) primary gastric epithelial cells isolated from gastric tissue, guinea pig fibroblasts CRL-1405 ATTC (purchased in American Type Culture Collection, Rockville, Manassas, VA, USA), were cultured and passaged as previously described^13–16^. Moreover, human THP-1 monocytes (ATCC TIB-202) or modified THP1-XBlue cells (purchased in Invitrogen, San Diego, CA, USA), were used. Human THP-1 cells were grown in Roswell Park Memorial Institute (RPMI-1640) medium supplemented with 10% heat-inactivated fetal calf serum (FCS), 100 U/mL penicillin, 100 U/mL streptomycin, 2 mM/mL l-glutamine at 37 °C, (all from Biowest, Nuaillé, France), in a humid atmosphere containing 5% CO_2_. THP1-XBlue cells were maintained in complete RPMI-1640 (cRPMI-1640) medium (Sigma Aldrich, Saint Louis, MO, USA), containing 10% FCS, with antibiotics: penicillin (100 IU/mL), streptomycin (100 μg/mL) (all in Biowest, Nuaillé, France), and selective agents: normocin (100 µg/mL) and blastocidin (10 µg/mL) (Invitrogen, San Diego, CA, USA), at 37 ℃ in a humidified atmosphere.

All cells were exposed for 24 h to blank MPs: CHI, CHI-Plur or CHI-GlcNAc at concentrations of 10 mg/mL and 5 mg/mL. As a positive control, standard *E. coli* lipopolysaccharide (LPS) (serotype O55:B5, Sigma Aldrich, St. Louis, Missouri, USA) was used at the concentration of 1 µg/mL. After stimulation we assessed: cell viability monocyte activation, DNA damage and cell apoptosis.

### Cell viability assay

Biocompatibility in vitro of blank MPs: CHI, CHI-Plur or CHI-GlcNAc at the concentration of 10 mg/mL or 5 mg/mL, was tested using the (3-(4,5-dimethylthiazol-2-yl)-2,5-diphenyltetrazolium bromide) (MTT) reduction assay according to the ISO norm 10993–5 (Biological evaluation of medical devices-Part 5: Tests for in vitro cytotoxicity; International Organization for Standardization, 2009) as previously described^31^. For this purpose, we used recommended L929 mouse fibroblasts, guinea pig primary gastric epithelial cells or fibroblasts, and human THP-1 monocytes. Four independent experiments were carried out in triplicate for each experimental variant.

### DNA damage

Blank MPs: CHI, CHI-Plur or CHI-GlcNAc at the concentration of 10 mg/mL or 5 mg/mL, or *E. coli* LPS (as a positive control), were added to the cultures of guinea pig primary gastric epithelial cells or fibroblasts for 24 h. DNA damage was determined using the HCS DNA Damage Kit (Thermo Fisher Scientific, Waltham, MA, USA) as recommended by the manufacturer, as previously described^31^. Four independent experiments were performed in triplicate.

### Cell apoptosis

Primary gastric epithelial cells and guinea pig fibroblasts after exposure to blank MPs: CHI, CHI-Plur or CHI-GlcNAc at the concentration of 10 mg/mL or 5 mg/mL, or *E. coli* LPS (as a positive control), were immunohistochemically stained for the presence of early pro-apoptotic caspase 3 (CC3) or middle apoptosis stage caspase 9 (CC9) as well as late apoptosis protein carbamoyl-phosphate synthetase 2 aspartate transcarbamylase and dihydroorotase (CAD). The procedure of staining was performed using fluorescently labelled rabbit-specific primary antibodies (Santa Cruz Biotechnology, Dallas, TX, USA), and then anti-rabbit Alexa Fluor 488-IgG secondary antibodies (Invitrogen, Waltham, MA, USA), as previously described^31^. The amount of CC9 and CAD was determined by measuring fluorescence intensity at 495 nm excitation and 519 nm emission, using a SpectraMaxi3 reader (Molecular Devices, San Jose, CA, USA). Four independent experiments were carried out in triplicate for each experimental variant.

### Monocyte activation assay

The THP-1XBlue cells were placed in 96-well tissue culture plates (5 × 10^4^ cells/well) and incubated for 24 h at 37 °C in a humified atmosphere with blank MPs: CHI, CHI-Plur, CHI-GlcNAc at the concentration 10 mg/mL and 5 mg/mL. The cells in culture medium alone (cRPMI-1640) served as a negative control, whereas cells treated with *E. coli* LPS O55:B5 (1 μg/mL) were a positive control, as previously described^31^. The optical density at 620 nm was measured using a microplate reader SpectraMax i3 (Molecular Devices, San Jose, CA, USA). Four independent experiments were carried out in triplicate for each experimental variant.

### Isolation and stimulation of guinea pig bone marrow macrophages

The guinea pig bone marrow macrophages were isolated to complete RPMI-1640 culture medium (cRPMI) from tibias and femurs as previously described^29^. Cells were adjusted to the density of 5 × 10^6^ cells/mL in cRPMI and underwent stimulation with *M. bovis* BCG or live *H. pylori* reference strain CCUG17874 (Culture Collection, University of Gothenburg, Gothenburg, Sweden), which was cultured under microaerophilic conditions according to the previously described procedure^13^. Macrophages were stimulated with *M. bovis* BCG/*H. pylori* using the multiplicity of infection (MOI): 10:1. The procedure of macrophage stimulation was as follows: priming with *M. bovis* BCG 24 h and 5 days restimulation with *M. bovis* BCG; priming with *M. bovis* BCG for 24 h, 5 days restimulation with *H. pylori;* priming with *M. bovis* BCG for 24 h, 5 days restimulation with *H. pylori,* and additional 24 h restimulation with *M. bovis* BCG. Stimulated or unstimulated control cells were then examined for phagocytic activity in conjunction with the assessment of expression of cell activation marker CD11b as well as global DNA methylation.

### Phagocytosis

Bone marrow macrophages (5 × 10^6^ cells/mL) were applied to the wells of a 96-well plate (100 µL/well) and stimulated with *M. bovis* BCG or with live *H. pylori* as described above. Phagocytosis—engulfment was assessed using commercial fluorescently-labelled *E. coli* and trypan blue quenching solution as recommended by the manufacturer (Vybrant Phagocytosis Assay Kit, Thermo Fisher Scientific, Waltham, MA, USA). The intensity of phagocytosis was determined by measuring the fluorescence using a SpectraMaxi3 reader (Molecular Devicesat, San Jose, CA, USA) at 495 nm (excitation) and 515 nm (emission). Four independent experiments were carried out in triplicate for each experimental variant.

### Surface deposition of CD11b

The guinea pig bone marrow macrophages, after priming and restimulation, were prepared for staining with fluorescently labelled primary rabbit anti-CD11b antibodies (Thermo Fisher Scientific, Waltham, MA, USA), and the secondary goat anti-rabbit antibodies Alexa Fluor 488-labelled (Invitrogen, CA, USA), as previously described^31^. The fluorescence intensity was measured using a SpectraMax i3 reader (Molecular Devicesat, San Jose, CA, USA), at the wavelengths for Alexa Fluor 488 (excitation 495 nm, emission 519 nm). Four independent experiments were carried out in triplicate for each experimental variant.

### Isolation of macrophage DNA and estimation of its global methylation

The DNA was isolated from macrophages, which underwent priming or priming and restimulation as described above, according to the Genomic Mini DNA purification protocol (A&A Biotechnology, Gdansk, Poland). The efficiency and purity of DNA were verified spectrophotometrically by Nanophotometer Pearl (Implen, Westlake, USA), 100–125 ng/μL, and A 260/280 ratio = 1.6, respectively. DNA methylation enzyme‐linked immunosorbent assay (ELISA; Epigenetek, Farmingdale, USA), with high DNA affinity strip wells, and capture as well as detection antibodies specific for 5hmC were used, as previously described^31^. To calculate the percentage of 5hmC in total DNA, the following formula was used: 5‐hmC% = (sample OD − negative control OD/slope × 100 ng) × 100%

### In vivo biocompatibility and biodistribution of tested microparticles

Biocompatibility and biodistribution of tested microparticles were assessed according to norm EN ISO 10993–1 “Biological evaluation of medical devices/Evaluation and testing in the risk management process/Biological evaluation process/Research in biological evaluation”.

### Ethical statements

All experiments involving animals were developed according to the ARRIVE guidelines and guidelines and regulations EU directive (Directive 2010/63/EU of the European Parliament and of the Council of 22 September 2010 on the protection of animals used for scientific purposes (Dz.U. L 276 z 20.10.2010, s. 33–79), and were approved by the Local Ethics Committee (LKE9) for Animal Experiments of the Medical University in Lodz, Poland, which was established by the Ministry of Science and Higher Education in Poland (ŁB 16/ 234/2022). Both genders of three-month old guinea pigs (five animals per group), free of pathogens, were bred and housed in the Animal House at the Faculty of Biology and Environmental Protection, University of Lodz (Poland). The animals were kept in air-conditioned rooms at 20–24 °C in cages with free access to drinking water and food pellets ad libitum. They were exposed to a 12 h light/dark cycle. The local and systemic deleterious effects of tested microparticles in vivo were tested in guinea pigs on two ways, after subcutaneous injection of MPs or after *per os* inoculation. The irritation potential of studied formulations, which may drive local and systemic inflammatory response, was excluded on the basis of skin reaction. For this purpose the animals were shaved from the dorsal area at the central site of the trunk 24 h before testing, then 0.2 mL of tested suspension of blank MPs: CHI, CHI-Plur, CHI-GlcNAc at the concentration of 10 mg/mL in 0.85% NaCl, prepared according to the norm PN EN ISO 10993–12:2012E, or diluent alone, were administered subcutaneously. The animals were monitored daily for water and food intake and behavioral symptoms. Skin reactions, defined as erythema and edema, were evaluated after 24, 48 and 72 h according to a skin reaction scoring system as previously described^31^. The appearance of edema and erythema was assessed and graded daily according to the Primary Irritation Index (PII) = 0/3, where 0 indicates that the irritation is negligible. After 72 h, the animals were euthanized with an overdose of sodium barbiturate (Morbital, Biowet, Puławy, Poland), and then the blood and organs (spleen, liver) were collected and examined to exclude tissue disorders or used for further testing.

Guinea pigs were inoculated *per os* with 1 mL of blank MPs: CHI, CHI-Plur, CHI-GlcNAc at the concentration of 10 mg/mL in 0.85% NaCl. Control animals received 1 mL of 0.85% NaCl*.* Nonpolar microparticles were prepared according to the norm PN EN ISO 10993–12:2012E. After 24, 48 and 72 h from inoculation with MPs, the animals were euthanized with an overdose of sodium barbiturate (Morbital, Biowet, Puławy, Poland), and then the blood and organs (spleen, liver) were collected and examined to exclude tissue disorders or used for further testing.

In all animals, the proliferative activity of spleen lymphocytes was determined in the presence of phytohemagglutinin (PHA) (Sigma-Aldrich, Saint Louis, MO, USA) or in culture medium alone (spontaneous proliferation). Proliferation assessment was performed based on the incorporation of radioactive thymidine—^3^H[dRT] (Moravec. Inc., Mercury Lane Brea, CA, USA) into the DNA of dividing cells during the last 18 h of cultivation, as previously described^13^. The concentration of alanine aminotransferase (ALT) and aspartate aminotransferase (AST) in liver homogenates and serum samples was determined by the commercial ELISA (MyBiosoure, San Diego, USA), with a sensitivity of 0.06 ng/mL and < 0.118 ng/mL, respectively, according to the attached protocol. Furthermore, the level of serum pro-inflammatory cytokines: tumor necrosis factor alfa (TNF-α) and interleukin (IL)-1B was examined by the ELISA (Thermo Fisher Scientific, Waltham, MA, USA), with the sensitivity 1.7 pg/mL (TNF-α) and 1 pg/mL (IL-1ß), respectively, as recommended by the manufacturer. Three independent experiments were performed in triplicate for each experimental variant.

### Statistical analysis

Data were expressed as median values ± range. The differences between groups were tested using the non-parametric *U* Mann–Whitney test. The Statistica 13 PL software (Kraków, Poland) was used for statistical analysis. Results were considered statistically significant when *p *< 0.05). To assess the distribution of normality, we used the Shapiro–Wilk test (S–W).

### Ethics approval and consent to participate

All experiments involving animals were developed according to the ARRIVE guidelines and guidelines and regulations EU directive (Directive 2010/63/EU of the European Parliament and of the Council of 22 September 2010 on the protection of animals used for scientific purposes (Dz.U. L 276 z 20.10.2010, s. 33–79) and were approved by the Local Ethics Committee (LKE9) for Animal Experiments of the Medical University in Lodz, Poland, which was established by the Ministry of Science and Higher Education in Poland (ŁB 16/ 234/2022). Both genders of three-month guinea pigs (five animals per group), free of pathogens, were bred and housed in the Animal House at the Faculty of Biology and Environmental Protection, University of Lodz (Poland).

**Methods**: preparation of chitosan microparticles by spray drying technique; characteristic of ingredients and chitosan-based microparticles loaded with *M. bovis* BCG; evaluation of the viability of *M. bovis* BCG encapsulated in chitosan microparticles; kinetics of *M. bovis* BCG release from chitosan microparticles in vitro and **Results:** characteristic of *M. bovis* BCG-loaded microparticles obtained by spray-drying technique; in vitro validation of cellular effects of unloaded chitosan microparticles; evaluation of the viability and kinetic of the release of *M. bovis* BCG encapsulated in chitosan microparticles in vitro*; *in vivo*,* biocompatibility and biodistribution of unloaded chitosan microparticles are the basis for the patent application number P.447595 to the Patent Office of the Republic of Poland.

## Results and discussion

### Influence of *M. bovis* BCG on engulfment properties of macrophages isolated from the bone marrow of *Cavia porcellus*

Previously we showed that *H. pylori* bacilli inhibit phagocytosis using their surface haemagglutinins or LPS^17,19,21^. Although these bacteria are not classified as typical intracellular pathogens they may survive inside phagocytes in megasomes^18^. In this study, in vitro experiments were established to see whether *M. bovis* BCG bacilli increase the engulfment capacity of *Cavia porcellus* bone marrow-derived macrophages (BMDM). Macrophages maintain homeostasis in the intestine and are involved in the development of immune processes in the gut^32,33^. It has been shown that in cell cultures of bovine monocytes in vitro* M. bovis* BCG bacilli stimulate these cells to the production of pro-inflammatory cytokines, including tumor necrosis factor alpha (TNF-α) and interleukin (IL)-6. In calves inoculated with *M. bovis* BCG aerosol mononuclear cells isolated from peripheral blood also responded by the enhanced production of pro-inflammatory cytokines^34^. In this study, we used a model of guinea pig BMDM as primary cells, which in cell culture in vitro undergo maturation^35^. These cells were also selected due to their compatibility with the in vivo* Cavia porcellus* model.

In this study, BMDM, as previously human THP-1 derived macrophages^29^, were treated for 24 h (induction-priming), with *M. bovis* BCG, and then the engulfment capacity of macrophages was evaluated. Furthermore, the influence of macrophage priming with mycobacteria on CD11b integrin expression and the level of total DNA methylation was determined. The effect of 5 days of BMDM restimulation with *M. bovis* BCG was also determined to investigate whether the cell response in these conditions is stronger. Furthermore, we followed the response of cells, which were first primed with mycobacteria and then re-exposed to *H. pylori* or restimulated additionally with *M. bovis* BCG for 24 h. This additional restimulation of cells with *M. bovis* BCG was used to see whether mycobacteria can upregulate macrophage phagocytic activity diminished by *H. pylori*. The phagocytic activity of BMDM was assessed using the reference Vybrant assay with *E. coli* fluorescently labelled.

Priming (24 h) of BMDM with mycobacteria resulted in an enhancement of *E. coli* engulfment as compared to such activity of cells propagated in culture medium alone (Fig. S1A,a). The *E. coli* ingestion intensity was similar when macrophages were restimulated for 5 days with homologous bacteria (*M. bovis* BCG) (Fig. S1A,b). However, it was lowered after restimulation of phagocytes with *H. pylori* for 5 days (Fig. S1A,c). Furthermore, additional restimulation (24 h) of macrophages with BCG did not increase the engulfment of *E. coli*, which was diminished in cells exposed to *H. pylori* (Fig. S1A,d). It has been revealed that *H. pylori* using different surface adhesins can block the phagocytes activity^21^. The mechanisms of mycobacteria, which can promote phagocytosis of different bacteria are unknown. In the case of mycobacteria, the cell surface bacterial components by binding phagocyte receptors drive the uptake of these bacilli by macrophages, which are the target niche for mycobacteria^36,37^. Thus phagocyte-mycobacteria interactions may potentially drive phagocytosis of other bacteria by macrophages, including *H. pylori*. It has been shown that phagocytosis of mycobacteria was initiated by THP-1 monocytes in response to the interaction of *M. tuberculosis* 19 kDa antigen with the mannose receptor^38^.

In the present work, a deposition of CD11b surface molecules on BMDM was evaluated (Fig. S1B). These molecules participate in transmigration and phagocytosis processes, including variants mediated by complement subunits, and the development of T cell-dependent tolerance^39^. BMDM treated with *M. bovis* BCG showed enhanced deposition of CD11b (Fig. S1Ba,b), which was then lowered after 5 days of exposure of macrophages to *H. pylori* (Fig. S1B,c). BMDM, which were treated additionally for 24 h with mycobacteria showed an elevated CD11b deposition (Fig. S1B,d). These results suggest that macrophages in which deposition of CD11b is temporarily inhibited by treatment of cells with *H. pylori* may be upregulated after cell restimulation with *M. bovis* BCG. These results reveal that the effects induced in macrophages by mycobacteria are flexible. The question arises whether the observed modulatory effects of *M. bovis* BCG towards macrophages are related to the innate memory mechanisms^40^.

Methylation of DNA is a biomarker of enhanced activity of monocytes and macrophages due to memory-like processes in innate immune cells^41–43^. BMDM, which were primed with *M. bovis* BCG or primed and then restimulated with *M. bovis* BCG, showed an increased DNA methylation (Fig. S1C,a,b,d). DNA methylation in BMDM pulsed with *M. bovis* BCG or restimulated with mycobacteria did not diminish in response to *H. pylori* (Fig. S1C,c). The preliminary results showing an upregulation of CD11b deposition on BMDM treated with BCG bacilli and enhanced engulfment capacity of macrophages suggest that epigenetic modulation may induce the above cell properties. Previously, we showed using *Cavia porcellus*, which were inoculated first with *M. bovis* BCG, and then with *H. pylori*, that the amount of mucin 5AC in the gastric tissue was diminished, and due to this the adhesion of *H. pylori* to gastric mucosa was lower^44^. We think that the binding of *M. bovis* BCG to gastric mucosa and upregulation by these bacilli of phagocytic activity of macrophages may potentially help to eradicate *H. pylori *in vivo*.* Thus, the obtained results prompt further studies on the effects of *per os* application of *M. bovis* BCG to *Cavia porcellus* with experimental *H. pylori* infection. Taking into account the above future application of *M. bovis* BCG in animal model, we developed CHI-MPs modified with Pluronic F-127 or with GlcNAc dedicated for upregulation of immune response towards *H. pylori*.

### Characteristic of *M. bovis* BCG-loaded microparticles obtained by spray-drying technique

The results showing the immunomodulatory properties of *M. bovis* BCG bacilli using *Cavia porcellus* BMDM prompted us to develop chitosan-based biocompatible microparticles resistant to the pH in the stomach or the colon and effectively encapsulate *M. bovis* BCG bacilli using such MPs. The combination of CHI and Pluronic F-127 allows preparing particles that are sensitive to pH^45^, or temperature fluctuations^46^, while GlcNAc enhances anti-*H. pylori* effect of *M. bovis* BCG^47^. Moreover, saccharides^48^ or polymers^49^ protect bacteria during the procedure of spray-drying.

The first step towards this aim was the analysis of used materials by CP/MAS NMR, as shown in Fig. S2A. CHI spectrum exhibits typical peaks at 174 (C=O signals), CH signals 105 (C1), 83.29 (C4), 75.42 (C5–C3), 61.18 (C6), 57.58 (C2), and 23.71 (CH_3_ signals) ppm, similarly as showed by Marcondes et al.^50^. Similarly, the spectrum of *N*-Acetyl-d-Glucosamine, a repeating unit of CHI, exhibits signals originating from carbonyl (175.12 ppm), CH signals (93.05, 74.40, 71.39, 70.26, 59.94, 52.42 ppm) and CH_3_ signal (24.48 ppm), as showed by Rajamohanan et al.^51^ Pluronic F-127 spectrum displayed defined signals of –CH_2_–CH– in the PPO block (75.68 and 73.77 ppm) and –CH_2_– in the PEO block (71.03 ppm), whereas the signal at 18 ppm originates from –CH_3_ in the PPO block^52^.

Subsequently, the spray drying technique was used to prepare blank and *M. bovis* BCG-loaded microparticles. Three sets of microparticles were prepared composed solely of CHI, a mixture of CHI with GlcNAc, or CHI with Pluronic F-127. The polymeric solutions form a clear solution (250 mL) in 1% acetic acid, and a good suspension was obtained after the addition of *M. bovis* BCG, which was suitable for the spray-drying process. The microparticles were used without additional purification steps. The yield of microparticles ranged from 10 to 43%, depending on the formulation.

Furthermore, the thermal properties of spray-dried microparticles were investigated. In the first heating run, two broad peaks are present around 102.2 and 162.6 °C for CHI-based microparticles. This effect is correlated with water evaporation, as reported by Villegas-Peralta et al. 2021 and it was confirmed by TGA analysis (Fig. S3A)^53^. Moreover, the melting temperature (*T*_m_) was not seen in the first heating run and also in the second heating run since *T*_m_ is close to the thermal decomposition process^54^, as shown in Fig. S3B). In the case of GlcNAc, the most important feature is its melting point around 205–210 °C; however, above this temperature, the decomposition occurs in the first heating run (Fig. S3B) as confirmed by TGA analysis (Fig. S3A). The characteristic Pluronic F-127 peaks are observed^55^, such as *T*_m_ in the first and second run around 54–56 °C and crystallization peak at 31 °C during cooling, as shown in Fig. S3B. The thermal analysis of CHI-GlcNAc and CHI-Pluronic microparticles indicates the water evaporation and melting of GlcNAc or Pluronic in the first heating run; moreover, in the second heating run of DSC, the melting of Pluronic F-127 crystallites is also showed (Fig. S3B).

We also analyzed *M. bovis* BCG IR spectrum, which we have divided into windows (W): W1 3000–2800 cm^−1^—fatty acids; W2 1800–1500 cm^−1^—peptides and proteins, W3 1500–1200 cm^−1^—proteins, phosphate carrying compounds and fatty acids, W4 1200–900 cm^−1^—carbohydrates and W5 900–750 cm^−1^—a unique fragment, the so-called “fingerprint” (Fig. S4)^56^.

The cell wall of mycobacteria contains unique carbohydrate and lipid complexes. The mycolic acids are the most variable lipid compounds, which show characteristic bands between 2850–2920 cm^−1^—unique for *M. bovis* BCG in the infrared spectra^57^. Carbonyl ester groups in lipids corresponded to the band^56^ at 1745 cm^−1^. The carbohydrate components of mycobacterial cell wall are arabinogalactan, arabinomannan, lipoarabinomannan, phosphatidylinositol mannosides, glycolipids and glucans. The “polysaccharide region” from 900 to 1200 cm^−1^ was useful for FT-IR characteristics due to the high content of arabinogalactan esterified with mycolic acids, which is bound with the peptidoglycan layer^56,58^. We also identified typical mycobacteria spectral regions containing the following prominent absorption peaks: 2920 cm^−1^ and 2850 cm^−1^ (W1), 1651 cm^−1^ and 1539 cm^−1^ (W2), 1462 cm^−1^, 1396 cm^−1^ and 1238 cm^−1^ (W3) as well as 1084 cm^−1^ (W4) (Fig. S4) In addition, FTIR was used to analyze blank (Fig. S2B) and *M. bovis* BCG-loaded microparticles prepared via spray drying (Fig. S2C). The chitosan microparticles show characteristic signals: from 3600 cm^−1^ to 3100 cm^−1^ (stretching vibration of O–H), 2900 cm^−1^ (the stretching vibration), 1543 cm^−1^ (the N–H bending and the C–N stretching vibrations), 1065 cm^−1^ (C=O stretching vibration). The low intensity of a band at 1647 cm^−1^, indicates that during the procedure of spray-drying the N-deacetylation may occur including GlcNAc^59^. There was no marked difference between the spectra of chitosan due to their structural similarity. In the contrary, two new bands appeared after the addition of Pluronic F-127 at 1342 cm^−1^ (CH_2_ wag) and 842 cm^−1^ (CH_2_ rock, C–O–C stretch)^57^. Also, in *M. bovis* BCG-loaded microparticles we detect lipid compounds unique for *M. bovis* BCG—characteristic signals from 2850 cm^−1^ to 2920 cm^−1^ (Fig. S2C).

**Figure 1:**
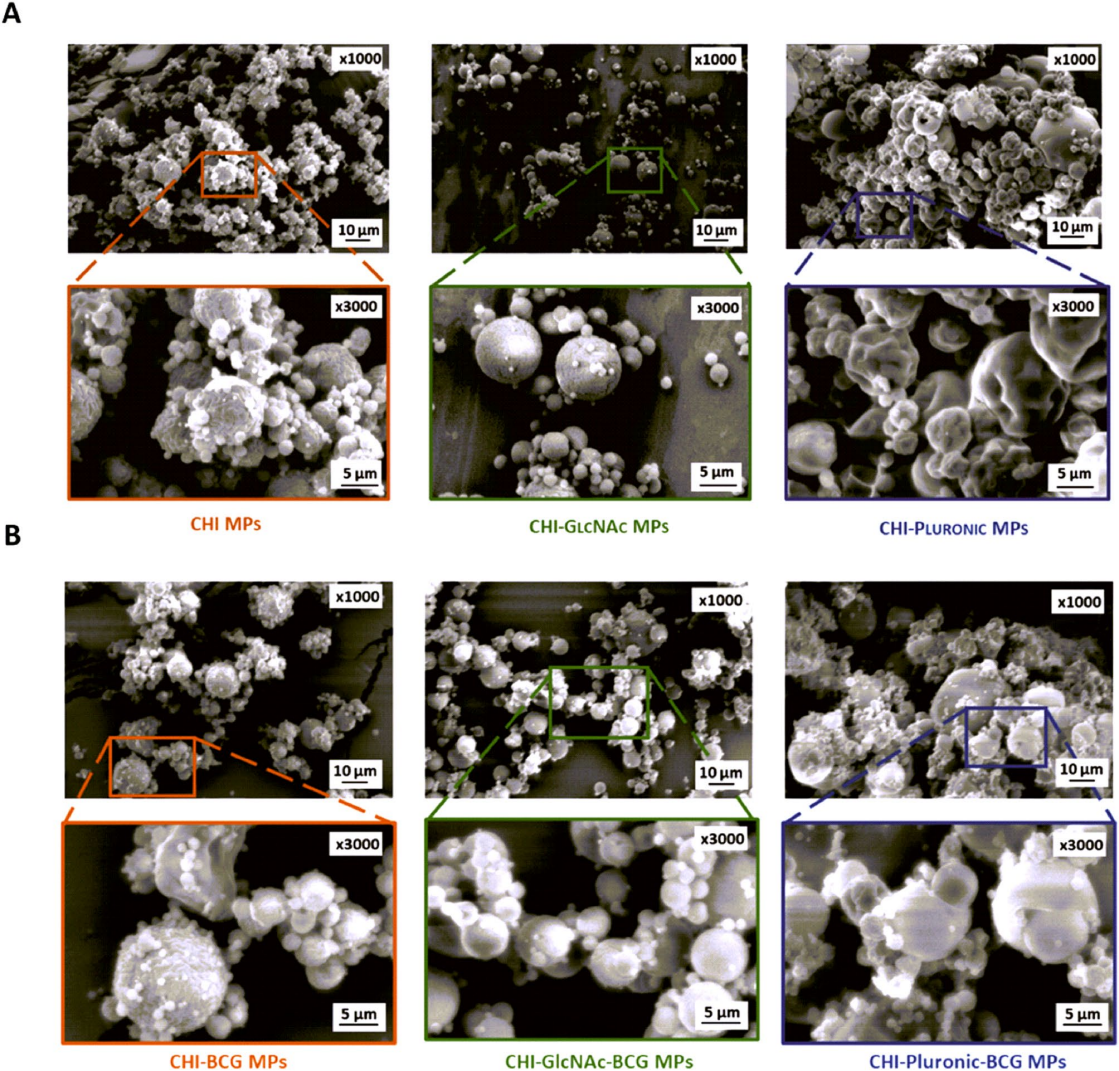
SEM microphotographs of blank chitosan microparticles (CHI MPs), CHI MPs modified with *N*-acetyl-d-glucosamine (CHI-GlcNAc MPs), and CHI MPs modified with Pluronic F-127 (CHI-Pluronic MPs) (**A**) and of CHI-BCG, CHI-GlcNAc-BCG, and CHI-Pluronic-BCG MPs (**B**).

The microparticles were designed to ensure a high loading of *M. bovis* BCG and simultaneously protection during their path through a gastrointestinal tract. The SEM analysis revealed that studied MPs exhibit a spherical shape with size heterogeneity in the range of 1 to 20 µm (Fig. 1A), similarly as showed by Reich et al.^60^. The presence of additives did not affect the size of developed microparticles, whereas the addition of Pluronic F-127 caused a slight collapse of their structure, similar to the report of Reich et al.^60^. Interestingly, the addition of *M. bovis* BCG to a formulation composed of CHI and GlcNAc improved their size distribution, and the collapse was no longer visible for the formulation with Pluronic F-127, as shown in Fig. 1B. In addition, the *M. bovis* BCG bacilli were not visible on the surface of the particles, which can be an indication of their successful encapsulation.

### Evaluation of the viability and release of *M. bovis* BCG encapsulated in chitosan microparticles in vitro

Microencapsulation of bacteria is mainly used for probiotic microorganisms to prevent them from acidic pH, the influence of toxic substances and storage conditions^61,62^. In this study for *M. bovis* BCG microencapsulation, we used pure CHI and CHI modified by *N*-Acetyl-d-glucosamine (GlcNAc) or surfactant Pluronic F-127 (Plur). GlcNAc addition to the CHI solution was dictated by its anti-*H. pylori* properties^63,64^, while Plur protects MPs against dissolution in gastric juice, which has a low pH^65^. Multivalent properties of CHI, such as mucoadhesive, antimicrobial, and mechanical properties, which prevent bacterial cell lysis, cause chitosan to meet the requirements for the preparation of microspheres^66,67^.

**Figure 2:**
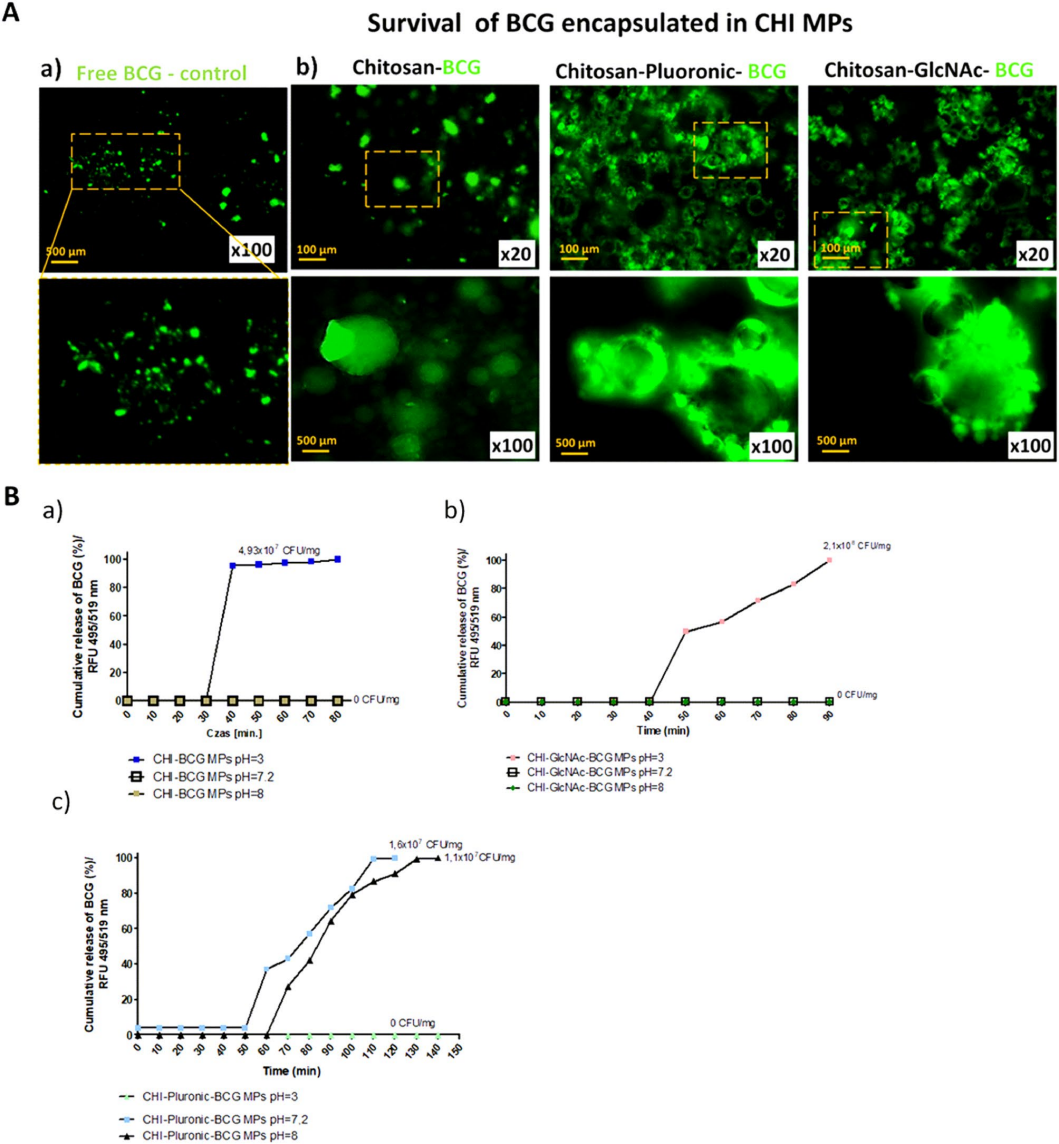
Survival of *M. bovis* in chitosan microparticles after spray drying process (**A**) and evaluation of release of *M. bovis* BCG encapsulated in chitosan microparticles at different pH (**B**). (**a**) *M. bovis* BCG (BCG) viability control—a representative image of the Bactligt stained *M. bovis* BCG in the fluorescent microscope (Zeiss, Axio Scope.A1, Zeiss, Jena, Germany) before adding chitosan (CHI) to the loading solution, at the wavelength 495 nm (excitation) and 519 nm (emission), at magnification ×100, under immersion. (**b**) representative image of live *M. bovis* BCG fluorescently labelled and encapsulated in CHI-MPs (Chitosan-BCG) by spray drying in a fluorescent microscope (Zeiss, Axio Scope.A1, Zeiss, Jena, Germany) at the wavelength 495 nm (excitation) and 519 nm (emission) at magnification ×20, ×100, under immersion. *M. bovis* BCG was stained using the BactLight staining procedure. Chitosan-Pluronic-BCG—chitosan microparticles modified with Pluronic F-127 and loaded with *M. bovis* BCG; Chitosan-GlcNAc-BCG—chitosan microparticles modified with *N*-Acetyl-d-Glucosamine and loaded with *M. bovis* BCG. Magnification ×20 or ×100. (**B**) The release of the *M. bovis* BCG from chitosan microparticles (CHI-MPs) was assessed at pH: 3.0, 7.2 and 8.0 every 10 min, at 495/519 nm. The fluorescently labelled *M. bovis* BCG alone were used as a control. The following CHI-MPs loaded with fluorescently labelled *M. bovis* BCG were used: non-modified CHI-MPs loaded with *M. bovis* BCG (CHI -BCG MPs), CHI-MPs with Pluronic F-127 loaded with *M. bovis* BCG (CHI-Pluronic-BCG MPs); CHI-MPS with *N*-Acetyl-d-Glucosamine loaded with *M. bovis* BCG (CHI-GlcNAc-BCG MPs). The fluorescence intensity was measured using a SpectraMaxi3 reader (Molecular Devices, San Jose, CA, USA) at 495 nm (excitation) and 519 nm (emission). Data are presented as median values of colony forming units (CFU)/mg ± range of four separate experiments (four independent experiments in triplicate for each experimental variant).

Our research showed that the process of spray drying of CHI solutions ensured the viability of *M. bovis* BGG in MPs (Fig. 2A). The viability of mycobacteria encapsulated in CHI-MPs was confirmed microbiologically after 6 weeks by spreading of bacteria on Lowenstein–Jensen agar according to the routine procedure.

Live *M. bovis* BCG encapsulated in CHI-MPs were released from specific MPs as follows: from CHI-*M. bovis* BCG MPs after 30–80 min, in pH 3.0 in a maximum quantity equal to 4.93 × 10^7^ CFU/mg in time 80 min (Fig. 2B) and from CHI-GlcNAc-*M. bovis* BCG MPs after 40–90 min, in pH 3.0 in a maximum quantity equal to 2.1 × 10^8^ CFU/mg in time 80 min (Fig. 2B). *M. bovis* BCG encapsulated in CHI-MPs were not released in pH = 8.0 or pH = 7.2, suggesting that CHI-MPs can release live *M. bovis* BCG in vivo in acidic pH in the stomach. *M. bovis* BCG bacilli are resistant to acidic pH due to urease production, which hydrolyzes urea into carbon dioxide and ammonia. Urease also protects mycobacteria against intracellular degradation^68^. In several studies, microencapsulation in pure CHI MPs of different bacterial species has been performed. For instance, encapsulated *Klebsiella* spp., are used for the removal of nitrogen from wastewater^69^, or *Lactobacillus casei*, as an addition to yoghurt^70^.

Live *M. bovis* BCG encapsulated in CHI-Plur MPs were released after 60–140 min, in pH 8.0 in a maximum quantity equal to 1.1 × 10^7^ CFU/mg in time 140 min, whereas in pH 7.2 were released after 50–110 min, in a maximum quantity equal 1.6 × 10^6^ CFU/mg in time 110 min (Fig. 2B). *M. bovis* BCG encapsulated in CHI-Plur MPs were not released in pH = 3.0, which suggests that in vivo CHI-Plur MPs loaded with *M. bovis* BCG can release live mycobacteria in the intestine where pH is alkaline.

Moreover, the release patterns of *M. bovis* BCG differ depending on the particle composition. The highest burst release was observed for CHI-based particles, followed by CHI-GlcNAc and CHI-Plur, respectively. This is caused by the presence of the Plur macromolecules on the surface of the particles^71^, which causes the decrease in burst release. This means that firstly bacteria present on the surface or loosely bonded to the particles diffuse to the surrounding media. The most favorable release profiles that exhibit CHI-GlcNAc and CHI-Plur for which the bacteria are released during the following minutes almost linearly; however, the longest release is observed for CHI-Plur MPs, indicating the positive effect of Plur on the overall process. We may only anticipate that the mechanism of their release can be a correlation of the diffusion or the erosion of the CHI matrix^72,73^.

### Evaluation of cellular effects of chitosan carrier

According to the ISO norm 10993–5:2009, all biomaterials for medical applications must fulfil the in vitro cytocompatibility criteria.

**Figure 3:**
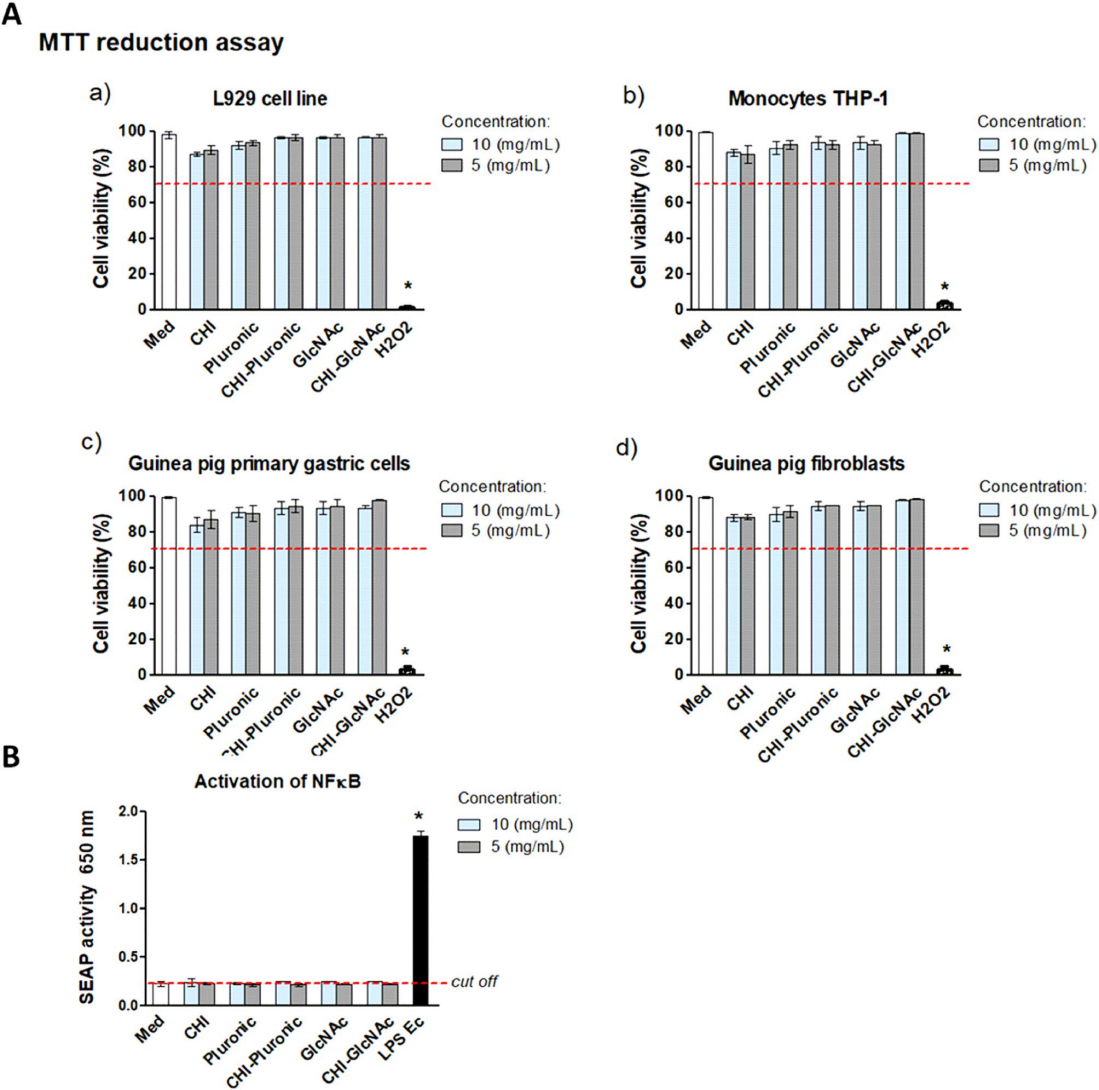
In vitro biosafety of blank MPs: CHI, CHI-Plur, CHI-GlcNAc in: (**A**) MTT reduction assay viability of (**a**) mouse fibroblasts L929, (**b**) human THP-1 monocytes, (**c**) primary gastric cells of *Cavia porcellus* (guinea pig), (**d**) guinea pig fibroblasts and (**B**) activation of nuclear factor kappa B in THP1-Blue monocytes.

For the assessment of cell biosafety of blank MPs: CHI, CHI-Plur and CHI-GlcNAc, we performed the reference MTT reduction assay and checked DNA damage in cells, in conjunction with apoptosis signs. These assessments were performed using L929 mouse fibroblasts (reference cells, recommended by ISO norm), primary guinea pig gastric cells and human THP-1 monocytes. As shown in Fig. 3A the viability of L929 cells (Fig. 3Aa), human THP-1 monocytes (Fig. 3Ab) or *Cavia porcellus* primary gastric cells (Fig. 3Ac) and fibroblasts (Fig. 3Ad) were not changed significantly (cell viability exceeded 70%) in cell cultures containing blank MPs: CHI, CHI-Plur, CHI-GlcNAc*.*

CHI may induce an inflammatory response of macrophages as a result of the induction of intracellular signaling pathways involving cyclic GMP–AMP synthase (cGAS), which is a stimulator of interferon genes (STING), and nod-like receptor (NLR) family pyrin domain 3 (NLRP3)^74–76^. We evaluated the ability of blank MPs: CHI, CHI-Plur, and CHI-GlcNAc to induce nuclear factor (NF)-kappa B signaling pathway in THP1-Blue monocytes by measurement of secreted alkaline phosphatase (SEAP) (Fig. 3B), which is exacerbated by LPS *E. coli* which was a positive control. This test allows the exclusion of a strong proinflammatory effect of tested components used for encapsulation^75^. Food and Drug Agency Guidance as well as the European Medicines Agency, recommend that the amount of endotoxin in material in contact with living tissue cannot be higher than 0.25 EU. We showed that SEAP concentration was comparable in control cell cultures and cell cultures containing tested MPs (Fig. 3B).

**Figure 4:**
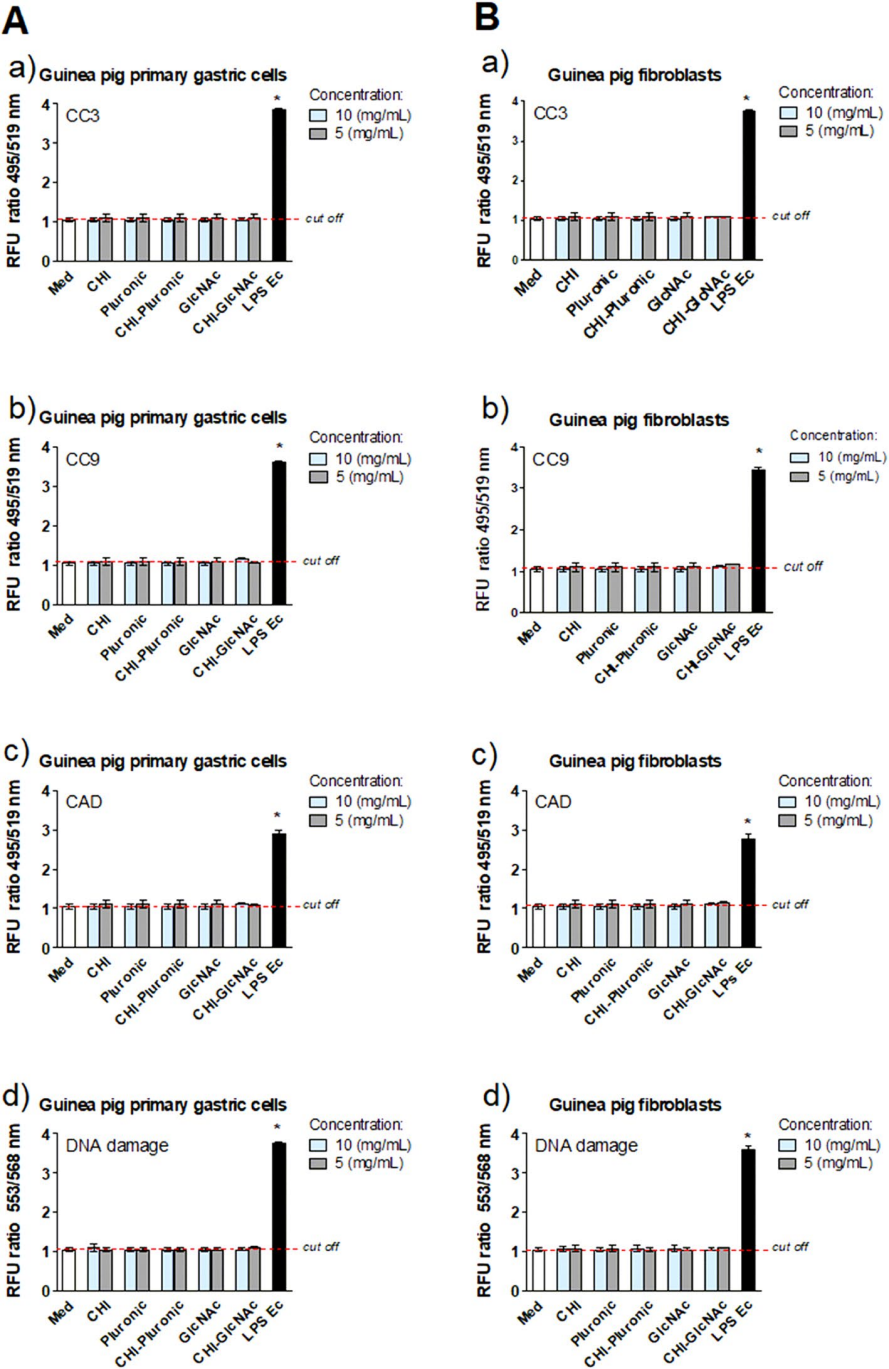
Determinants of apoptosis and DNA damage in cells exposed to unloaded MPs: CHI, CHI-Plur, CHI-GlcNAc. Apoptosis was assessed in primary gastric cells of *Cavia porcellus* (guinea pig) (**A**) or fibroblasts (**B**) on the basis of the measurement of fluorescence (**a**) caspase 3—CC3, (**b**) caspase 9—CC9 and (**c**) carbamoyl-phosphate synthetase 2 aspartate transcarbamylase and dihydroorotase—CAD. DNA damage (**d**) was assessed by measurement of fluorescence of phosphorylated pH2AX in gastric cells (**A**) or fibroblasts (**B**). Microparticles (MPs) were used at the concentration of 10 mg/mL and 5 mg/mL. Lipopolysaccharide (LPS) of *E. coli* (1 µg/mL) was a positive control (LPS Ec). The intensity of fluorescence was measured in a SpectraMax i3 reader (Molecular Devices, San Jose, CA, USA). The results are shown in relative fluorescence units (RFU). *p < 0.05; *control cells (Med) vs. cells exposed to tested microparticles (MPs). Data are shown as median ± range from four independent experiments in triplicate for each experimental variant. Statistical significance: CHI-chitosan, Pluronic-Pluronic F-127, GlcNAc–*N*-Acetyl-d-Glucosamine.

Apoptosis is a programmed cell death, which eliminates defective host cells^77–79^. However, excessive apoptosis may lead to dysfunction of different organs^79^. We tested whether blank MPs: CHI, CHI-Plur, and CHI-GlcNAc do not elevate apoptosis of studied cells by examination the CC3 level, CC9 level and CAD. None of the tested CHI-MPs promote apoptosis Fig. 4A, a,b,c and B, a,b,c.

It was consistent with that unloaded MPs: CHI, CHI-Plur, CHI-GlcNAc did not induce DNA damage assessed on the basis of phosphorylated gamma H2A.X (phospho-Ser139) vs. control cells (Fig. 4Ad, B,d). Fernandes et al*.* revealed no genotoxic effect of CHI towards lymphocytes^80^ while Jena et al. did not show the genotoxic activity of CHI-coated silver nanoparticles using a model of mouse macrophages (RAW264.7 cells)^81^.

The cell viability was determined on the basis of reduction by metabolically active cells of 3-(4,5-dimethylthiazol-2-yl)-2,5-, diphenyltetrazolium bromide (MTT). 100% of viable cells in complete RMPI-1640 culture medium (cRPMI) was a positive control (Med) while 100% of dead cells treated with 0.03% H_2_O_2_ was a negative control (H_2_O_2_). Cells were treated with MPs 10 mg/mL or 5 mg/mL. The red line shows 70% of viable cells required to exclude cytotoxicity of tested biomaterial in vitro. Statistical significance: *p < 0.05; *control cells vs*.* cells in cultures with tested microparticles (MPs). The results are shown as median ± range of four independent experiments in triplicate for each experimental variant. MPs—microparticles, CHI-chitosan, Pluronic-Pluronic F-127, GlcNAc–*N*-Acetyl-d-Glucosamine. Activation of nuclear factor kappa B (NF-kappa B) was evaluated in cells incubated for 24 h with unloaded MPs: CHI, CHI-Pluronic, CHI-GlcNAc at the concentration of 10 mg/mL and 5 mg/mL on the basis of secreted embryonic alkaline phosphatase (SEAP). Cells in culture medium alone (Med) were used as a negative control, while monocytes stimulated with lipopolysaccharide (LPS) of *E. coli* (LPS Ec) served as a positive control. Data are presented as median values ± range of four separate experiments (four independent experiments in triplicate for each experimental variant). Statistical significance: *p < 0.05; *untreated cells and cells treated with tested MPs or cells treated with LPS Ec. CHI-chitosan, Pluronic-Pluronic F-127, GlcNAc–*N*-Acetyl-d-Glucosamine.

### Assessment of in vivo biosafety of blank chitosan microparticles

Particles for medical usage must be checked for biosafety in vivo^82,83^. In the present study, the guinea pigs were used for assessment of the biosafety of tested MPs: CHI, CHI-Plur, and CHI-GlcNAc, without *M. bovis* BCG bacilli.

We check local cutaneous toxicity of tested MPs after subcutaneous injection using the primary irritation index to exclude sensitization and pro-inflammatory potential of tested MPs. The skin effects may be an indirect marker of potential local effects of tested MPs on the gastric epithelium as well as systemic. We also assessed systemic effects of tested MPs after *per os* inoculation of animals.

**Figure 5:**
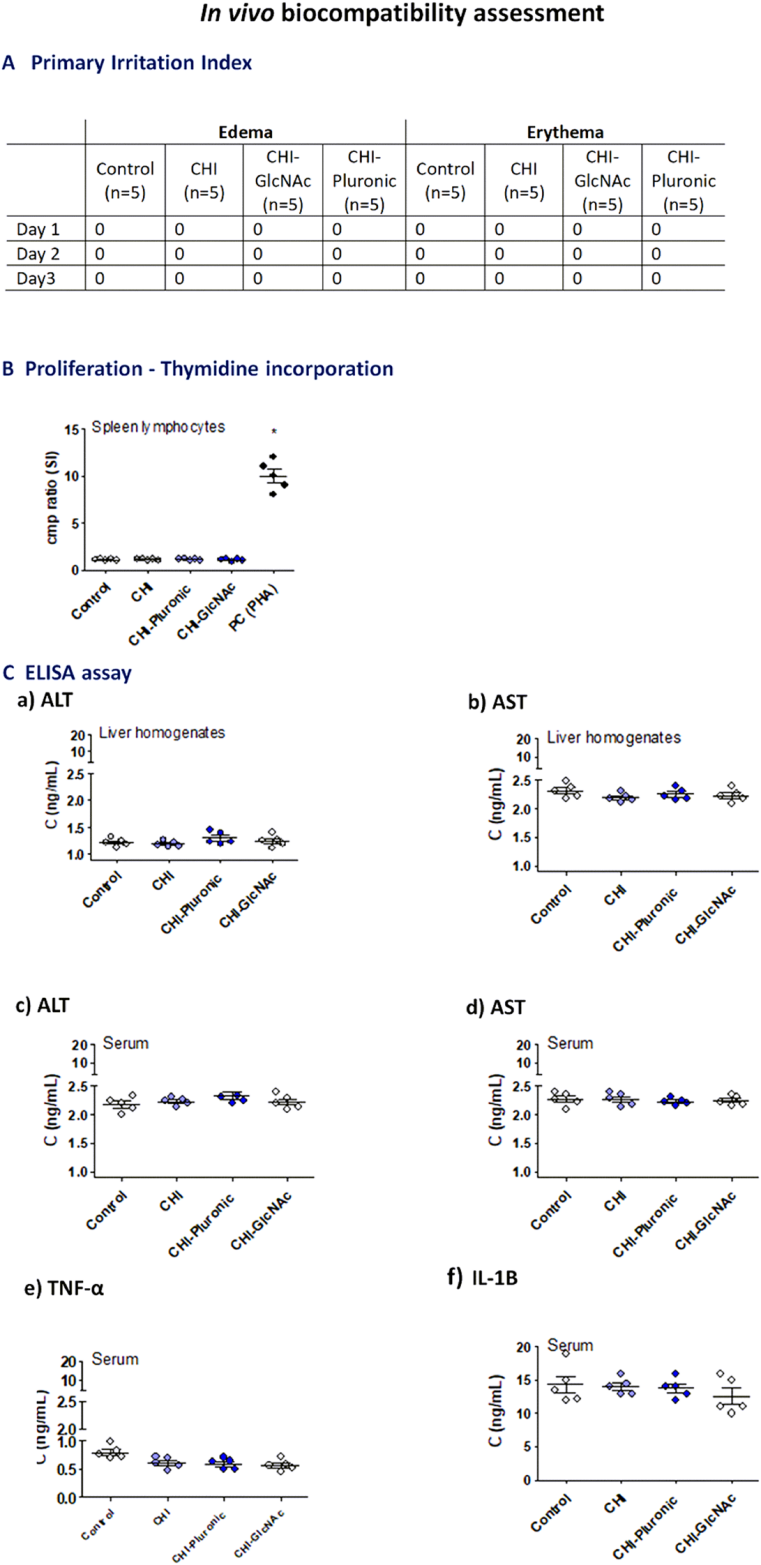
Biosafety of tested microparticles in vivo. Chitosan microparticles (CHI), chitosan microparticles with Pluronic F-127 (CHI-Pluronic), chitosan microparticles with *N*-Acetyl-d-Glucosamine (CHI-GlcNAc) were used. Guinea pigs were injected subcutaneously with tested CHI MPs or 0.85% NaCl and observed for the development of deleterious skin reactions (**A**). Edema and erythema were examined and graded daily according to the Primary Irritation Index (PII) = 0/3, negligible irritation—0. After 72 h after injection of MPs the animals underwent euthanasia, for isolation of spleen, liver and blood samples (**B**). Splenocytes were tested for proliferation activity in the presence of positive control (PC)—phytohemagglutinin (PHA) by (^3^H)-thymidine incorporation assay. Stimulation index (SI) was assessed: radioactivity counts (cpm/min) of cells treated with CHI-MPs/radioactivity of untreated cells. (**C**) The level of alanine aminotransferase ALT (**a**) or aspartate aminotransferase AST (**b**) in homogenized liver tissue or serum (**c**,**d**); the level of tumor necrosis factor-alpha—TNF-α (**e**) and interleukin (IL)-1ß (**f**) in serum (ELISA). Five animals were in the group. The results are shown as median ± range.

There were no signs of local skin irritation or erythema in animals after subcutaneous injection of MPs: CHI, CHI-Plur, and CHI-GlcNAc (Fig. 5A). In animals injected with MPs the signs of inflammation in the spleen were determined together with proliferation of splenocytes. Inflammatory markers and the level of splenocyte proliferation in animals receiving MPs were similar to these markers in control animals (Fig. 5B). In homogenates of liver tissue from control animals or MPs receiving guinea pigs also the levels of alanine aminotransferase (ALT) (Fig. 5C,a) or aminotransferase (AST) (Fig. 5C,b) were comparable. Furthermore, there was no difference between ALT and AST in serum samples (Fig. 5C,c and C,d), in animals injected with blank MPs and control animals. Moreover, in tested and control animals there was no difference in the serum level of TNF-α (Fig. 5C,e) or IL-1 B (Fig. 5C,f).

**Figure 6:**
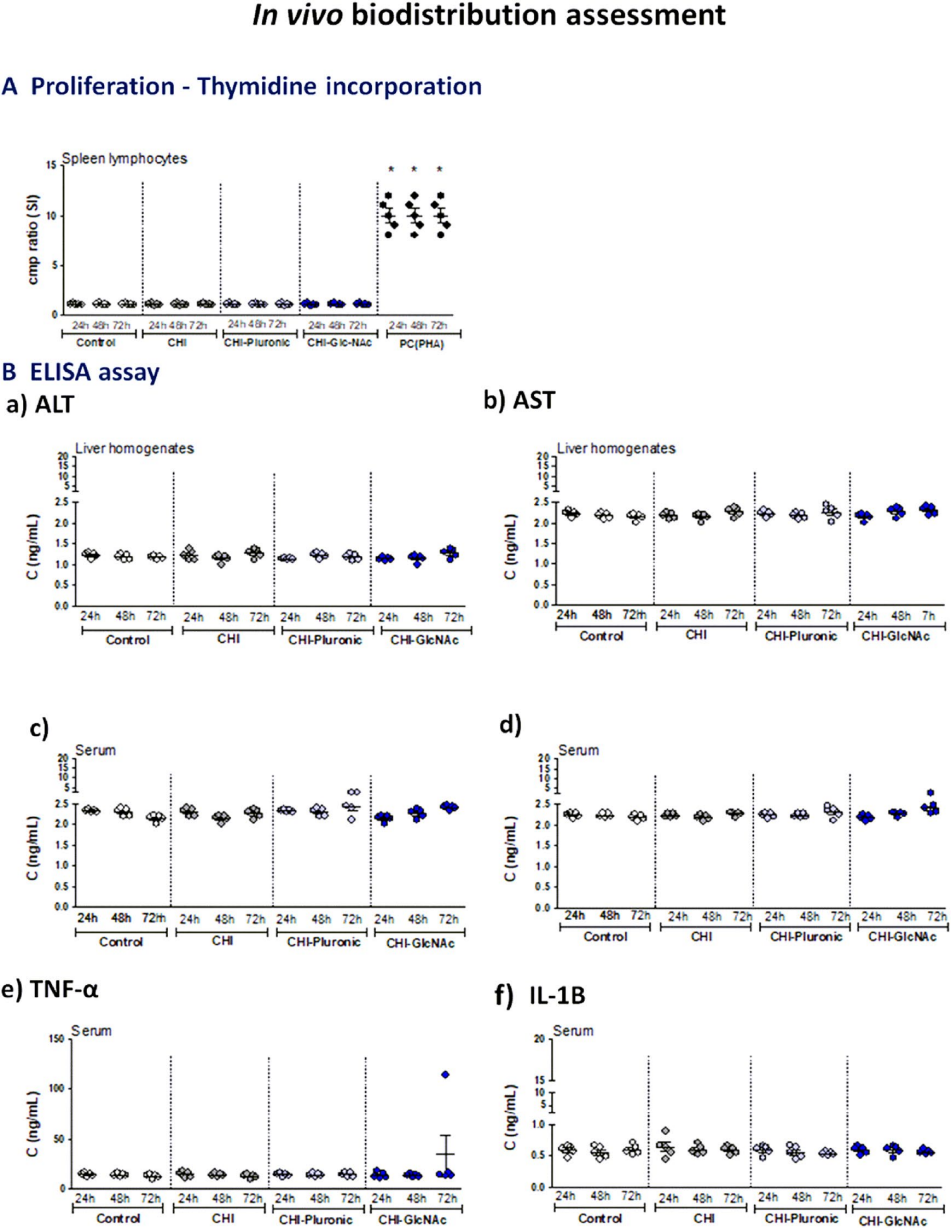
Biodistribution of tested blank chitosan microparticles in guinea pigs inoculated *per os*. Chitosan microparticles (CHI-MPs); chitosan microparticles with Pluronic F-127 (CHI-Pluronic), chitosan microparticles with *N*-Acetyl-d-Glucosamine (CHI-GlcNAc). Animals receiving *per os* tested MPs or 0.85% NaCl (control animals), were terminated for isolation of tissue samples (spleen, liver) or the collection of blood (**A**) Splenocytes were tested for proliferation in response to phytohemagglutinin (PHA), which was a positive control (PC) by (^3^H)-thymidine incorporation. Stimulation index (SI): radioactivity counts (cpm/min) of cells exposed to tested MPs/radioactivity of control cells (**B**) The level of alanine aminotransferase ALT (**a**) and aspartate aminotransferase AST (**b**) in liver tissue homogenates or serum (**c**,**d**); the level of tumor necrosis factor alfa (TNF-α) (**e**) or interleukin (IL)-1ß (**f**) in serum (ELISA). Five animals were in the group. The results are shown as median ± range.

After *per os* inoculation of animals with blank CHI-MPs or such MPs modified with Plur (CHI-Plur MPs) or with GLcNAc (CHI-GlcNAc) the proliferation of splenocytes was determined (Fig. 6A), and ALT and AST were assessed in liver tissue homogenates (Fig. 6Ba,b) or serum (Fig. 6Bc,d). Also, the serum level of TNF-α or IL-1 B (Fig. 6Be,f) was evaluated in animals receiving tested MPs. In animals receiving tested MPs the levels of the above biomarkers were similar to those in control animals.

Pandey and Khuller showed that in guinea pigs receiving trice blank alginate/chitosan microparticles (125 mg/kg each dose), there were no toxic effects showed as no difference in serum level of bilirubin, ALT or AST^84^. Also, nanoparticles based on chitosan/poly-γ-glutamic acid were not toxic after *per os* administration of 100 mg/kg, daily for 14 days as showed by the lack of histopathological signs in the liver, kidney and in intestine and no alteration of ALT and AST. After intranasal administration to mice of CHI-graft-spermine/pDNA nanoparticles, there was no induction of hemorrhage or inflammation. The authors did not show disorders in brain, heart, lung, liver, kidney and spleen^85,86^.

## Conclusions

The ability of macrophages to phagocytosis of the reference bacteria *E. coli* and CD11b expression were both diminished by *H. pylori. Mycobacterium bovis* BCG bacilli upregulated the phagocytic activity of macrophages obtained from guinea pig bone marrow in vitro. The elevated cell phagocytic activity provoked by *M. bovis* BCG was related to increased deposition of CD11b and global DNA methylation. We have developed fully biocompatible carriers—CHI-MPs, loaded with *M. bovis* BCG, ensuring that mycobacteria are predominantly inside the carrier and released at a specific pH, acidic or alkaline. These MPs are dedicated to improving the treatment procedures for *H. pylori* infections. This is due to the bactericidal properties of CHI against these bacteria^87–89^, as well as *M. bovis* BCG immunomodulatory properties. Therefore, it is possible to use *M. bovis* BCG encapsulated in CHI-MPs in vivo in the *Cavia porcellus* model with experimental *H. pylori* infection to confirm the ability of mycobacteria to support the immune response against *H. pylori*. The model of *Cavia porcellus* was characterized by us in terms of an immune response, which develops after inoculation of animals with *H. pylori*; thus, this model is optimal^90^.

### Supplementary Information

Supplementary Figures.

## Data Availability

All data generated or analyzed during this study are included in this published article or supplementary file. The datasets used and/or analyzed during the current study are available from the corresponding author on reasonable request.

## References

[1] Davis ME (2008). Nanoparticle therapeutics: An emerging treatment modality for cancer. Nat. Rev. Drug Discov..

[2] Kean T, Thanou M (2010). Biodegradation, biodistribution and toxicity of chitosan. Adv. Drug Deliv. Rev..

[3] Chander S, Hasnain MS, Beg S, Nayak AK (2022). Chapter 4—Role of chitosan in transdermal drug delivery. Chitosan Drug Delivery.

[4] Sicard J-F (2017). Interactions of intestinal bacteria with components of the intestinal mucus. Front. Cell. Infect. Microbiol..

[5] Safer AM, Leporatti S (2021). Chitosan nanoparticles for antiviral drug delivery: A novel route for COVID-19 treatment. Int. J. Nanomed..

[6] Sharifi-Rad J (2021). Chitosan nanoparticles as a promising tool in nanomedicine with particular emphasis on oncological treatment. Cancer Cell Int..

[7] Herdiana Y (2022). Drug release study of the chitosan-based nanoparticles. Heliyon..

[8] Nayak AK, Hasnain MS, Beg S, Nayak AK (2022). Chapter 3—Chitosan-based nanoparticles in drug delivery. Chitosan Drug Delivery.

[9] Dey S, Hasnain MS, Beg S, Nayak AK (2022). Chapter 11—Cross-linking of chitosan in drug delivery. Chitosan Drug Delivery.

[10] Shukla R, Hasnain MS, Beg S, Nayak AK (2022). Chapter 17—Chitosan for delivery of biomolecules. Chitosan Drug Delivery.

[11] Warren JR, Marshall B (1983). Unidentified curved bacilli on gastric epithelium in active chronic gastritis. Lancet.

[12] Chmiela M, Kupcinskas J (2019). Pathogenesis of *Helicobacter pylori* infection. Helicobacter..

[13] Gonciarz W (2019). The effect of *Helicobacter*
*pylori* infection and different *H.*
*pylori* components on the proliferation and apoptosis of gastric epithelial cells and fibroblasts. PLoS One.

[14] Mnich E (2016). Impact of *Helicobacter pylori* on the healing process of the gastric barrier. World J. Gastroenterol..

[15] Gonciarz W (2020). Proregenerative activity of IL-33 in gastric tissue cells undergoing *Helicobacter pylori*-induced apoptosis. Int. J. Mol. Sci..

[16] Gonciarz W (2021). Interference of LPS *H*. *pylori* with IL-33 driven regeneration of *Cavia*
*porcellus* primary gastric epithelial cells and fibroblasts. Cells.

[17] Chmiela M (1997). Role of *Helicobacter pylori* surface structures in bacterial interaction with macrophages. Gut.

[18] Allen LA (2007). Phagocytosis and persistence of *Helicobacter pylori*. Cell Microbiol..

[19] Grębowska A (2008). Anti-phagocytic activity of *Helicobacter pylori* lipopolysaccharide (LPS)—Possible modulation of the innate immune response to these bacteria. Pol. J. Microbiol..

[20] Rudnicka K (2015). *Helicobacter pylori*-driven modulation of NK cell expansion, intracellular cytokine expression and cytotoxic activity. Innate Immun..

[21] Paziak-Domańska B (2002). Potential role of CagA in the inhibition of T cell reactivity in *Helicobacter pylori* infections. Cell Immunol..

[22] Savoldi A (2018). Prevalence of antibiotic resistance in *Helicobacter*
*pylori*: A systematic review and meta-analysis in World Health Organization regions. Gastroenterology.

[23] Cui R (2021). Analysis among genotype resistance, phenotype resistance and eradication effect of *Helicobacter pylori*. Infect. Drug Resist..

[24] Bujanda L (2021). Antibiotic resistance prevalence and trends in patients infected with *Helicobacter*
*pylori* in the period 2013–2020: Results of the European registry on *H*. *pylori* management (hp-Eureg). Antibiotics.

[25] Freyne B (2015). BCG-associated heterologous immunity, a historical perspective: Intervention studies in animal models of infectious diseases. Trans. R. Soc. Trop. Med. Hyg..

[26] Aaby P (2011). Randomized trial of BCG vaccination at birth to low-birth-weight children: Beneficial nonspecific effects in the neonatal period?. J. Infect. Dis..

[27] Wardhana DE (2011). The efficacy of Bacillus Calmette-Guerin vaccinations for the prevention of acute upper respiratory tract infection in the elderly. Acta Med. Indones..

[28] Hana J (2020). Mechanisms of BCG in the treatment of bladder cancer-current understanding and the prospect. Biomed. Pharmacother..

[29] Gonciarz W (2023). *Mycobacterium bovis* BCG increase the selected determinants of monocyte/macrophage activity, which were diminished in response to gastric pathogen *Helicobacter pylori*. Sci. Rep..

[30] Narayanan VHB (2022). Spray-dried tenofovir alafenamide-chitosan nanoparticles loaded oleogels as a long-acting injectable depot system of anti-HIV drug. Int. J. Biol. Macromol..

[31] Gonciarz W (2023). Stereocomplexed microparticles loaded with *Salvia*
*cadmica* Boiss. extracts for enhancement of immune response towards *Helicobacter*
*pylori*. Sci. Rep..

[32] Bain CC, Schridde A (2018). Origin, differentiation, and function of intestinal macrophages. Front. Immunol..

[33] Jaumouillé V, Waterman CM (2020). Physical constraints and forces involved in phagocytosis. Front. Immunol..

[34] Guerra-Maupome M (2019). Aerosol vaccination with Bacille Calmette-Guerin induces a trained innate immune phenotype in calves. PLoS One.

[35] Barrett JP (2015). Bone marrow-derived macrophages from aged rats are more responsive to inflammatory stimuli. J. Neuroinflamm..

[36] Squeglia F (2017). Structural overview of mycobacterial adhesins: Key biomarkers for diagnostics and therapeutics. Protein Sci..

[37] Ernst JD (1998). Macrophage receptors for *Mycobacterium tuberculosis*. Infect. Immun..

[38] Diaz-Silvestre H (2005). The 19 kD antigen of *Mycobacterium tuberculosis* is a major adhesin that binds the mannose receptor of THP-1 monocytic cells and promotes phagocytosis of mycobacteria. Microb. Pathog..

[39] Ehirchiou D (2007). CD11b facilitates the development of peripheral tolerance by suppressing Th17 differentiation. J. Exp. Med..

[40] Rusek P (2018). Infectious agents as stimuli of trained innate immunity. Int. J. Mol. Sci..

[41] Keating ST, El-Osta A (2015). Epigenetics and metabolism. Circ. Res..

[42] Yang Y (2018). PSTPIP2 connects DNA methylation to macrophage polarization in CCL4-induced mouse model of hepatic fibrosis. Oncogene.

[43] Ji J (2019). Methionine attenuates lipopolysaccharide-induced inflammatory responses via DNA methylation in macrophages. ACS Omega.

[44] Gonciarz W (2023). Diminishing of *Helicobacter pylori* adhesion to *Cavia porcellus* gastric epithelial cells by BCG vaccine mycobacteria. Sci. Rep..

[45] Sudipta C (2019). Dual-responsive (pH/temperature) Pluronic F-127 hydrogel drug delivery system for textile-based transdermal therapy. Sci. Rep..

[46] Chitosan F (2010). Synthesis and characterization of thermally responsive pluronic controlled release and intracellular delivery of small molecules. ACS Nano.

[47] Kawakubo M (2004). Natural antibiotic function of a human gastric mucin against *Helicobacter pylori* infection. Science.

[48] Sunny-Roberts EO, Knorr D (2009). The protective effect of monosodium glutamate on survival of *Lactobacillus*
*rhamnosus* GG and *Lactobacillus rhamnosus* E-97800 (E800) strains during spray-drying and storage in trehalose-containing powders. Int. Dairy J..

[49] de Lodato Se Govia P (1999). Viability and thermal stability of a strain of *Saccharomyces*
*cerevisiae* freeze-dried in different sugar and polymer matrices. App. Microbiol. Biotechnol..

[50] Marcondes W (2020). Evaluation of chitosan crystallinity: A high-resolution solid-state NMR spectroscopy approach. Carbohydr. Polym..

[51] Rajamohanan PR (1996). State CP/MASS 13C-NMR spectroscopy: A sensitive method to monitor enzymatic hydrolysis of chitin. J. Biochem. Biophysical Meth..

[52] Oc HH, Yoon KB (2008). Solid-state NMR study on the structure and dynamics of triblock copolymer P123 remaining in SBA-15 after solvent washing. Bull. Korean Chem. Soc. B..

[53] Villegas-Peralta Y (2021). Impact of the molecular weight on the size of chitosan nanoparticles: Characterization and its solid-state application. Polym. Bull..

[54] Acosta-Ferreira S (2020). Production and physicochemical characterization of chitosan for the harvesting of wild microalgae consortia. Biotechnol. Rep..

[55] Karolewicz B (2017). Pluronic F127 as a suitable carrier for preparing the imatinib base solid dispersions and its potential in development of a modified release dosage forms: Thermal, spectroscopic, microscopic, and dissolution studies. J. Therm. Anal. Calor..

[56] Naumann D, Meyers RA (2000). Infrared spectroscopy in microbiology. Encyclopedia of Analytical Chemistry.

[57] Wenning M (2006). Rapid analysis of two foodborne microbial communities at the species level by Fourier-Transform Infrared microspectroscopy. Environ. Microbiol..

[58] Lee RE (2005). Rapid structural characterization of the arabinogalactan and lipoarabinomannan in live mycobacterial cells using 2D and 3D HR-MAS NMR: Structural changes in the arabinan due to ethambutol treatment and gene mutation are observed. Glycobiology.

[59] Priya Dharshini K (2021). pH-sensitive chitosan nanoparticles loaded with dolutegravir as milk and food admixture for paediatric anti-HIV therapy. Carbohyd. Polym..

[60] Reich S (2019). High-temperature spray-dried polymer/bacteria microparticles for electrospinning of composite nonwovens. Macromol. Biosci..

[61] Atia A (2017). Molecular and biopharmaceutical investigation of alginate–inulin synbiotic coencapsulation of probiotic to target the colon. J. Microencapsul..

[62] Misra S (2021). The approaches for co-encapsulation of probiotic bacteria with bioactive compounds, their health benefits and functional food product development: A review. Trends Food Sci. Technol..

[63] Ferreira B (2006). Terminal α1, 4-linked *N*-acetylglucosamine in *Helicobacter pylori-*associated intestinal metaplasia of the human stomach and gastric carcinoma cell lines. J. Histochem. Cytochem..

[64] Lee H (2008). α1, 4GlcNAc-capped mucin-type O-glycan inhibits cholesterol α-glucosyltransferase from *Helicobacter*
*pylori* and suppresses *H*. *pylori* growth. Glycobiology.

[65] Chatterjee S (2019). Dual-responsive (pH/temperature) pluronic F-127 hydrogel drug delivery system for textile-based transdermal therapy. Sci. Rep..

[66] Serp D (2000). Characterization of an encapsulation device for the production of monodisperse alginate beads for cell immobilization. Biotechnol. Bioeng..

[67] Wu Q-X (2016). Evaluation of chitosan hydrochloride-alginate as enteric micro-probiotic-carrier with dual protective barriers. Int. J. Biol. Macromol..

[68] Vandal OH (2009). Acid resistance in *Mycobacterium tuberculosis*. J. Bacteriol..

[69] Zhao R (2020). Enhanced stability and nitrogen removal efficiency of *Klebsiella* sp. entrapped in chitosan beads applied in the domestic sewage system. RSC Adv..

[70] Enache IM (2020). Co-microencapsulation of anthocyanins from black currant extract and lactic acid bacteria in biopolymeric matrices. Molecules.

[71] Gong CY (2009). In vitro drug release behavior from a novel thermosensitive composite hydrogel based on Pluronic f127 and poly(ethylene glycol)-poly(ε-caprolactone)-poly(ethylene glycol) copolymer. BMC Biotechnol..

[72] Rao K-M (2023). pH sensitive drug delivery behavior of palmyra palm kernel hydrogel of chemotherapeutic agent. Gels.

[73] Bruschi ML (2015). Main mechanisms to control the drug release. Strategies to Modify the Drug Release from Pharmaceutical Systems.

[74] Bueter CL (2014). Spectrum and mechanisms of inflammasome activation by chitosan. J. Immunol..

[75] Carroll EC (2016). The vaccine adjuvant chitosan promotes cellular immunity via DNA sensor cGAS-STING-dependent induction of type I interferons. Immunity.

[76] Mantovani A (2013). Macrophage plasticity and polarization in tissue repair and remodelling. J. Pathol..

[77] Hotchkiss RS (2003). Role of apoptotic cell death in sepsis. Scand. J. Infect. Dis..

[78] Hengartner MO (2000). The biochemistry of apoptosis. Nature.

[79] Finkel E (2001). The mitochondrion: Is it central to apoptosis. Science.

[80] Fernandes JC (2011). Cytotoxicity and genotoxicity of chitooligosaccharides upon lymphocytes. Int. J. Biol. Macromol..

[81] Jena P (2012). Toxicity and antibacterial assessment of chitosan coated silver nanoparticles on human pathogens and macrophage cells. Int. J. Nanomed..

[82] Hoshyar N (2016). The effect of nanoparticle size on *in vivo* pharmacokinetics and cellular interaction. Nanomedicine.

[83] Drasler B (2017). In vitro approaches to assess the hazard of nanomaterials. NanoImpact.

[84] Pandey R, Khuller GK (2004). Chemotherapeutic potential of alginate-chitosan microspheres as anti-tubercular drug carriers. J. Antimicrob. Chemother..

[85] Sonaje K (2009). In vivo evaluation of safety and efficacy of self-assembled nanoparticles for oral insulin delivery. Biomaterials.

[86] Jiang H-L (2011). Chitosan-graft-spermine as a gene carrier in vitro and in vivo. Eur. J. Pharm. Biopharm..

[87] Luo D (2009). Preparation and evaluation of anti-*Helicobacter pylori* efficacy of chitosan nanoparticles in vitro and in vivo. J. Biomater. Sci..

[88] Nogueira F (2013). Effect of gastric environment on *Helicobacter pylori* adhesion to a mucoadhesive polymer. Acta Biomater..

[89] Henriques PC (2020). Orally administrated chitosan microspheres bind *Helicobacter pylori* and decrease gastric infection in mice. Acta Biomater..

[90] Walencka M (2015). The microbiological, histological, immunological and molecular determinants of *Helicobacter pylori* infection in guinea pigs as a convenient animal model to study pathogenicity of these bacteria and the infection dependent immune response of the host. Acta Biochim. Pol..

