# Supplementary material for: Spray-dried pH-sensitive chitosan microparticles loaded with Mycobacterium bovis BCG intended for supporting treatment of Helicobacter pylori infection
Source: Sci Rep. 2024 Feb 27;14:4747. doi: 10.1038/s41598-024-55353-6 (PMC10899647; doi:10.1038/s41598-024-55353-6)
Supplement: Supplementary file 1 — Supplementary Figures. [file 41598_2024_55353_MOESM1_ESM.docx]

Supporting Information for:

**Spray-dried pH-sensitive chitosan microparticles loaded with *Mycobacterium bovis* - BCG intended for supporting treatment of *Helicobacter pylori* infection**

Weronika Gonciarza, Marek Brzeziński, Weronika Orłowska, Paweł Wawrzyniak, Artur Lewandowski, Vedha Hari B. Narayanand, Magdalena Chmiela

**Table of Contents**

Figure S1. Increased phagocytic activity in conjunction with increased deposition of CD11b surface molecules as well as global DNA methylation in guinea pig bone marrow macrophages primed and then restimulated with *M. bovis* BCG *in vitro*.

Figure S2. (A) CP/MAS NMR spectrum of microparticles (MPs): chitosan (CHI), CHI- N-acetyl-D-glucosamine (GlcNAc), CHI- Pluronic F-127. (B) FTIR spectra of CHI, CHI-GlcNAc, and CHI-Pluronic MPs. (C FTIR spectra of CHI, CHI-GlcNAc, and CHI-Pluronic MPs loaded with *M. bovis* BCG.

Figure S3. A - TGA curves of microparticles (MPs): chitosan (CHI), CHI- N-acetyl-D-glucosamine(GlcNAc), CHI- Pluronic F-127, B- DSC thermograms of particles building blocks and the obtained particles.

Figure S4. Analysis of *M. bovis* BCG IR spectrum. A- Representative infrared spectra of *M. bovis BCG* W1–W4 correspond to the characteristic fragments of fatty acids (W1 3000–2800 cm− 1); peptides and proteins (W2 1800–1500 cm−1); proteins, phosphate-carrying compounds and fatty acids (W3 1500–1200 cm− 1); carbohydrates (W4 1200–900 cm− 1). The arrows indicate the unique wave numbers for BCG- B figure.


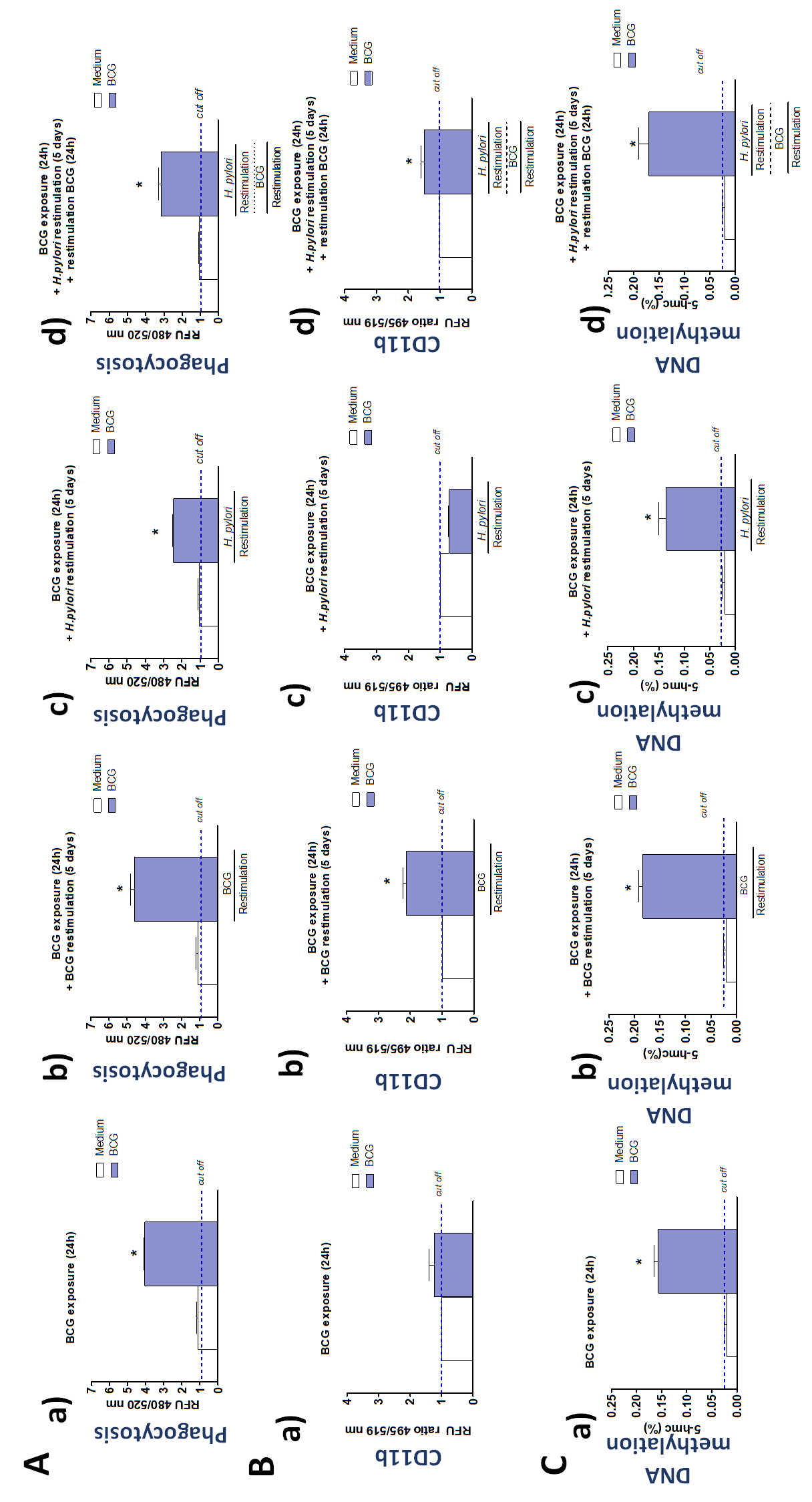


Figure S1. Increased phagocytic activity in conjunction with increased deposition of CD11b surface molecules as well as global DNA methylation in guinea pig bone marrow macrophages primed and then restimulated with *M. bovis* BCG *in vitro*. The guinea pig bone marrow-derived macrophages (BMDM) were treated with *M. bovis* BCG alone for 24 h (a), restimulated for 5 days with *M. bovis* BCG (b) or with *H. pylori* (c), and for additional 24 h with *M. bovis* BCG (d). **A)** Phagocytosis towards fluorescently labelled *Escherichia coli* was determined on the basis of the relative fluorescence units (RFU) ratio in relation to phagocytic activity of control cells in the culture medium alone. The results are presented as median RFU ratio ± range. **B)** Deposition of CD11b cell surface integrin was determined by the immunofluorescence staining of cells with anti-CD11b antibodies and showed as median RFU ratio ± range. The fluorescence intensity was measured in a Multiscan^EX^ reader at 495 nm (excitation) and 519nm (emission). **C)** The global DNA methylation was determined by the ELISA with high DNA affinity strip wells, and capture as well as detection antibodies specific for 5hmC were used. The quantity of hydroxymethylated DNA fragments was detected colorimetrically at OD=450 nm. The percentage of 5hmC in DNA samples was calculated in reference to the standard curve. Statistical significance for p <0.05 in the non-parametric *U* Mann-Whitney test. * cells treated with stimulators vs. control cells in culture medium alone; ● cells primed with *M. bovis* BCG (24h) vs. cells primed with *M. bovis* BCG (24 h) and restimulated with *M. bovis* BCG (5 days); # cells primed with *M. bovis* BCG (24h) vs. cells primed with *M. bovis* BCG (24 h), restimulated for 5 days with *H. pylori*; # cells primed with *M. bovis* BCG (24 h) vs. cells primed with *M. bovis* BCG (24 h), restimulated for 5 days with *H. pylori.*


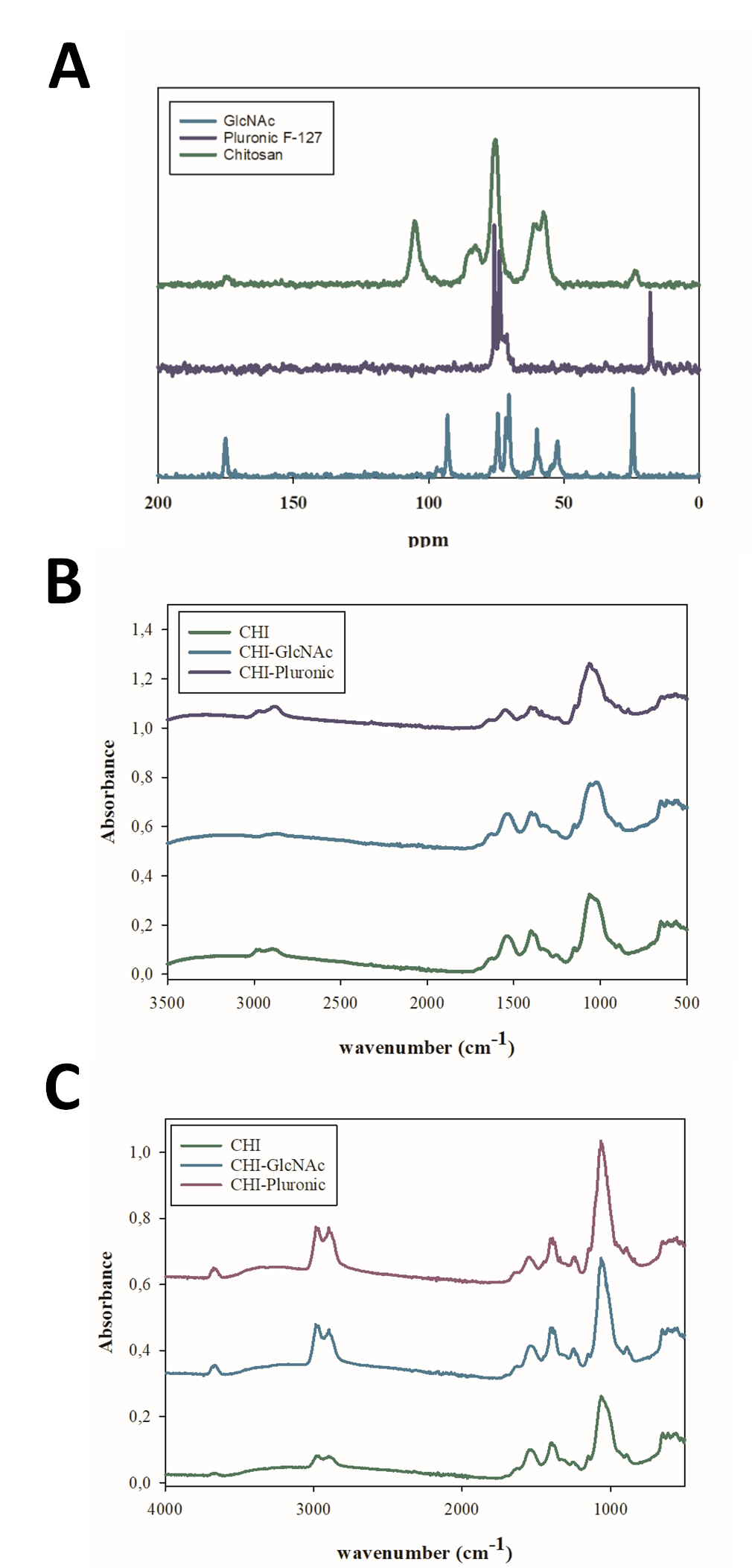


Figure S2. (A) CP/MAS NMR spectrum of microparticles (MPs): chitosan (CHI), CHI- N-acetyl-D-glucosamine(GlcNAc), CHI- Pluronic F-127. (B) FTIR spectra of chitosan (CHI) microparticles (MPs), chitosan microparticles modified with N-acetyl-D-glucosamine (CHI-GlcNAc), and chitosan microparticles modified with Pluronic F-127 (CHI-Pluronic). (C) FTIR spectra of chitosan microparticles (CHI), chitosan microparticles modified with N-acetyl-D-glucosamine (CHI-GlcNAc), and chitosan microparticles modified with Pluronic F-127 (CHI-Pluronic) loaded with *M. bovis* BCG.


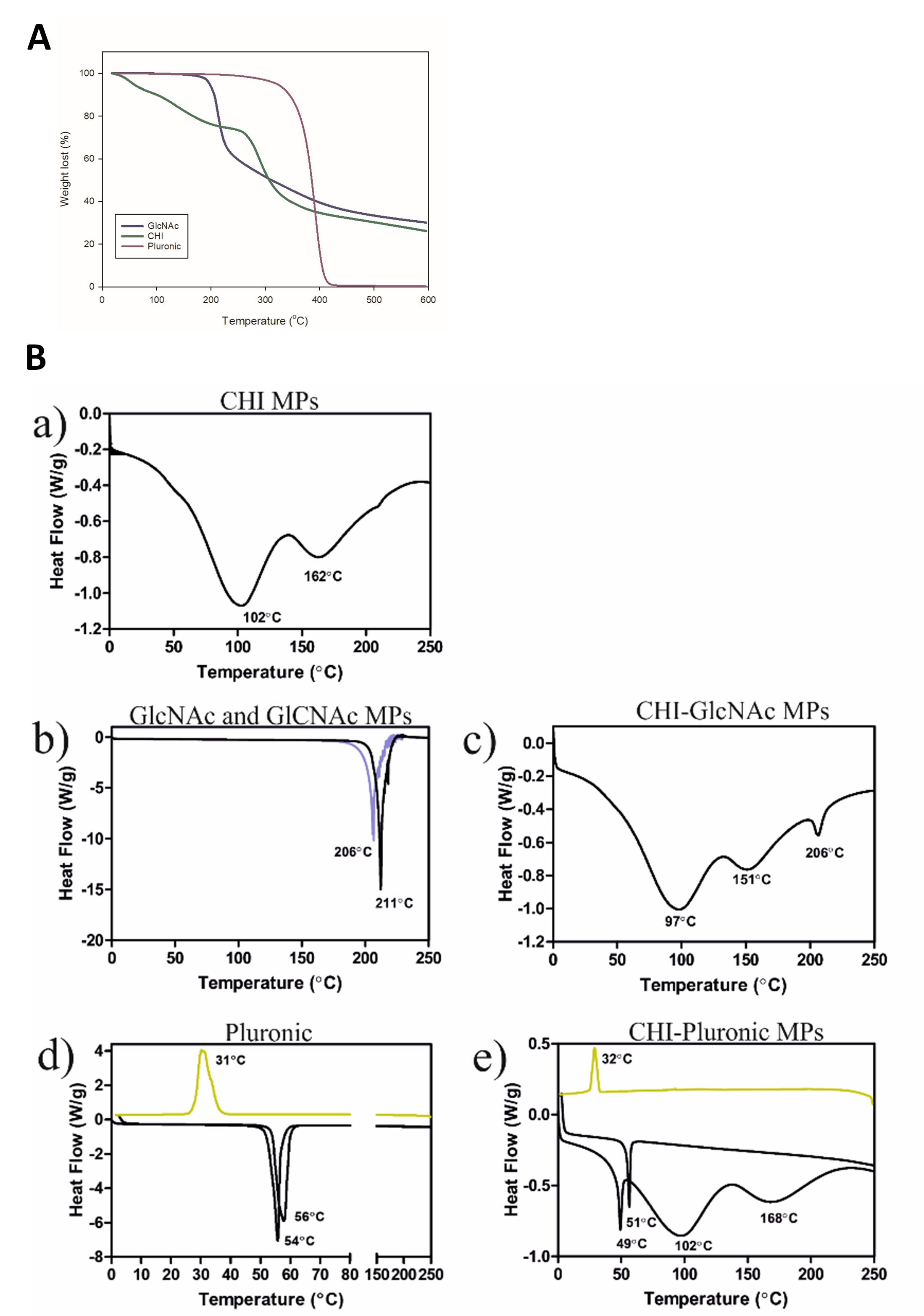


Figure S3. (A) TGA curves of spectrum of microparticles (MPs): chitosan (CHI) , CHI- N-acetyl-D-glucosamine(GlcNAc), CHI- Pluronic F-127 (Pluronic). (B) DSC thermograms of particles building blocks and the obtained particles. Chitosan microparticles (CHI-MPs), N acetyl-D-glucosamine (GLcNAc), Pluronic F-127 (Pluronic).


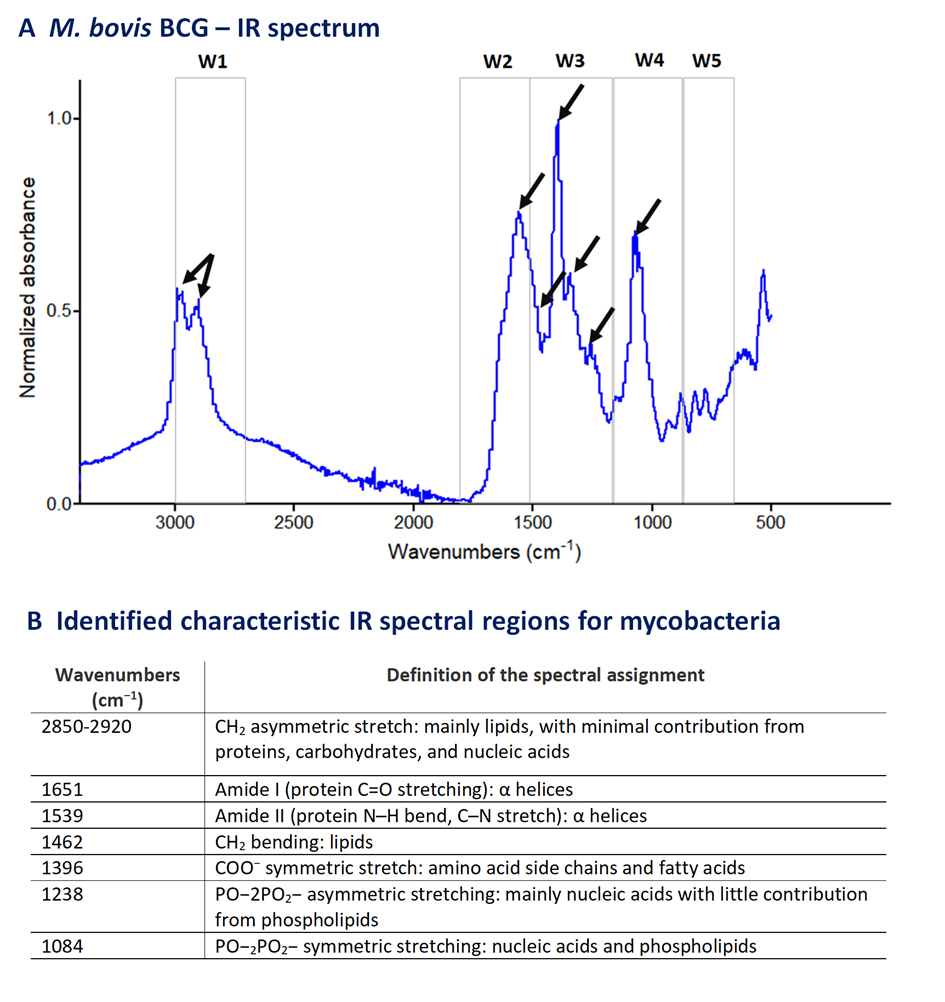


Figure S4. Analysis of *M. bovis* BCG IR spectrum. A- Representative infrared spectra of *M. bovis* BCG W1–W4 correspond to the characteristic fragments of fatty acids (W1 3000–2800 cm− 1); peptides and proteins (W2 1800–1500 cm−1); proteins, phosphate-carrying compounds and fatty acids (W3 1500–1200 cm− 1); carbohydrates (W4 1200–900 cm− 1). The arrows indicate the unique wave numbers for *M. bovis* BCG- B figure. Chitosan (CHI), N-acetyl-D-glucosamine (GlcNAc), Pluronic F-127 (Pluronic).
